# Breaking barriers: bacterial-microalgae symbiotic systems as a probiotic delivery system

**DOI:** 10.1186/s12951-024-02647-6

**Published:** 2024-06-25

**Authors:** Hui Huang, Xiaoyang Liu, Yutong Lang, Jiarong Cui, Danni Zhong, Min Zhou

**Affiliations:** 1 Eye Center, the Second Affiliated Hospital, Zhejiang University School of Medicine, Hangzhou, 310009 China; 2 Institute of Translational Medicine, Zhejiang University, Hangzhou, 310009 China; 3 Zhejiang University-University of Edinburgh Institute (ZJE), Zhejiang University, Haining, 314400 China; 4 State Key Laboratory (SKL) of Biobased Transportation Fuel Technology, Zhejiang University, Hangzhou, 310027 China

**Keywords:** Bacteria-microalgae symbiotic system, Probiotic delivery, Gut microbiota, *Spirulina platensis*, Inflammatory bowel disease

## Abstract

The gut microbiota is one of the essential contributors of the pathogenesis and progress of inflammatory bowel disease (IBD). Compared with first-line drug therapy, probiotic supplementation has emerged as a viable and secure therapeutic approach for managing IBD through the regulation of both the immune system and gut microbiota. Nevertheless, the efficacy of oral probiotic supplements is hindered by their susceptibility to the gastrointestinal barrier, leading to diminished bioavailability and restricted intestinal colonization. Here, we developed a bacteria-microalgae symbiosis system (EcN-SP) for targeted intestinal delivery of probiotics and highly effective treatment of colitis. The utilization of mircroalge *Spirulina platensis* (SP) as a natural carrier for the probiotic *Escherichia coli* Nissle 1917 (EcN) demonstrated potential benefits in promoting EcN proliferation, facilitating effective intestinal delivery and colonization. The alterations in the binding affinity of EcN-SP within the gastrointestinal environment, coupled with the distinctive structural properties of the SP carrier, served to overcome gastrointestinal barriers, minimizing transgastric EcN loss and enabling sustained intestinal retention and colonization. The oral administration of EcN-SP could effectively treat IBD by reducing the expression of intestinal inflammatory factors, maintaining the intestinal barrier and regulating the balance of gut microbiota. This probiotic delivery approach is inspired by symbiotic interactions found in nature and offers advantages in terms of feasibility, safety, and efficacy, thus holding significant promise for the management of gastrointestinal disorders.

**Supplementary Information:**

The online version contains supplementary material available at 10.1186/s12951-024-02647-6.

## Introduction

The gut microbiota, a vast and intricate assemblage of microorganisms, is frequently regarded as an acquired organ, commonly referred to as the “second brain” of the human body [1]. This microbial community plays a crucial role in maintaining the health of its host through various physiological functions, including bolstering intestinal integrity, regulating intestinal epithelial cell proliferation, supplying energy, preventing pathogen invasion, and modulating the host’s immunity [2, 3]. Research has demonstrated a strong correlation between human health and the proportion, structure and composition of gut microbiota [4]. Enteric dysbacteriosis has been identified as a potential contributor to a range of gastrointestinal and systemic diseases [5, 6]. Therefore, it is imperative to prioritize the maintenance of gut microbiota homeostasis in the prevention and treatment of human diseases. The adaptability of the gut microbiota allows for manipulation through external influences, such as probiotic supplementation [7, 8]. This treatment method is commonly utilized to regulate intestinal function, effectively restoring gut microbiota homeostasis by inhibiting pathogen colonization and safeguarding the integrity of the intestinal mucosal barrier [9–12]. Compared with fecal microbiota transplantation (FMT), oral delivery of probiotics to the gut microbiota represents a less invasive approach that is perceived as more acceptable and safer by patients [13, 14]. Oral administration of probiotics encounters significant obstacles including low viability, limited colonization, and inadequate functionality within the gastrointestinal tract [15]. Recent advancements have explored the utilization of various synthetic oral carriers for the effective encapsulation and transportation of probiotics. Nevertheless, these delivery carriers exhibit drawbacks including diminished biosafety, intricate design and construction, and limited potential for clinical translation. Therefore, developing safe, efficient, and convenient strategies for oral delivery of probiotics remains a formidable challenge.

Nature has inspired us with natural strategies for delivering probiotics, as evidenced by the symbiotic relationship between bacteria and algae that has been observed since the early stages of biological evolution [16, 17]. This symbiosis serves as a structural pillar of ecosystems and has practical applications in various fields, including industrial and municipal wastewater treatment [18, 19], carbon dioxide emission reduction, bioreactors, and energy production [20–22]. Nevertheless, limited research exists on the utilization of bacteria-microalgae symbiosis systems in the field of biomedicine. Microalgae, single-celled photosynthetic organisms predominantly located in oceans and freshwater lakes, exhibit rapid photosynthesis, growth, and high nutritional content. They have been extensively utilized in various applications such as sewage treatment, fuel production, food processing, and health supplements [23–27]. Recently, there has been a growing interest in utilizing microalgae as natural biomaterials in the biomedical field [28], showing significant benefits in biological imaging, drug delivery, hypoxic tumor therapy, wound healing [29–32]. According to the literature, microalgae contain various compounds with prebiotic properties, including galacto-oligosaccharides, inulin, arabinoxylans, and xylo-oligosaccharides [33, 34]. These cell wall polysaccharides can stimulate the growth and activity of beneficial bacteria [34–36]. Here, we pay attention to *Spirulina platensis* (SP), a microalga that is extensively cultivated for industrial purposes and has garnered recommendations from numerous organizations for its potential applications in medical, pharmaceutical, and nutritional supplements [37, 38]. SP has been found to possess anti-inflammatory, antioxidant, and gut microbiota-regulating properties [39, 40]. Furthermore, SP positively influences the viability of various probiotics, including *Lactobacillus bulgaricus*, *Lactobacillus acidophilus*, *Lactococcus lactis*, and *Streptococcus thermophilus* [36, 41]. Additionally, its favorable attributes, including good biocompatibility, cost-effectiveness, large active surface area, strong phototaxis, and significant propulsion force, have positioned SP as a promising candidate for natural drug delivery systems [42, 43]. In view of this, the utilization of microalgae-based bacterial drug delivery systems presents a novel approach for the administration of oral probiotics.

**Fig. 1:**
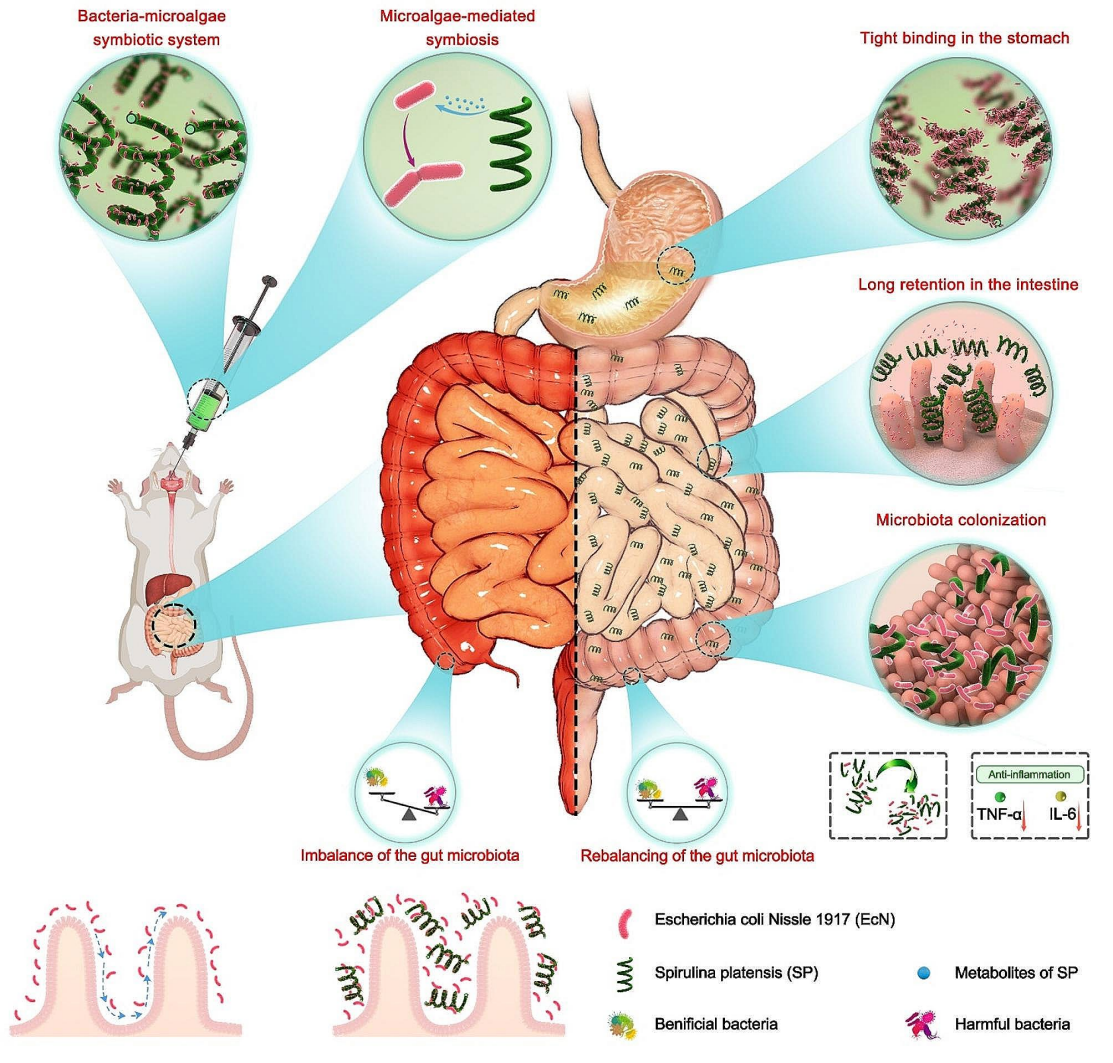
Schematic illustration of bacteria-microalgae symbiotic system (EcN-SP) for the high-efficient EcN delivery and treatment of inflammatory bowel disease. (i) In the stomach, EcN is closely bound to SP, and EcN-SP passes through the stomach quickly after oral administration. (ii) In the intestine, EcN-SP with helical morphology is easily captured by intestinal villi and stays in the intestine for a long time. (iii) EcN-SP gradually degrades in the intestine, releasing EcN and promoting its colonization. The bacteria-microalgae symbiotic system EcN-SP is effective in the treatment of colitis by down-regulating proinflammatory factors levels to reduce intestinal inflammation, and by promoting the abundance of probiotics and reducing the abundance of harmful bacteria to maintain the balance of gut microbiota

Here, we propose a natural delivery strategy utilizing a symbiotic relationship between bacteria and microalgae to facilitate the effective, safe, and environmentally friendly oral application of probiotics. Specifically, we employ SP as a natural carrier for delivering *Escherichia coli* Nissle 1917 (EcN), and the resulting bacteria-microalgae symbiosis system (EcN-SP) demonstrates enhanced efficiency in EcN delivery for the treatment of inflammatory bowel disease (IBD) (Fig. 1). EcN is a notable probiotic with proven efficacy in alleviating symptoms of ulcerative colitis [44, 45]. The presence of SP can enhance the living conditions for EcN and facilitate the proliferation of EcN within the gastrointestinal tract. The distinctive helical structure of ECN-SP allows for efficient capture by intestinal villi and adherence to the intestinal wall, thereby extending the co-delivery duration of EcN and SP in the intestine. After oral administration, EcN-SP demonstrates antioxidant and anti-inflammatory effects, thereby mitigating intestinal inflammation. The symbiotic relationship between SP and EcN demonstrates the potential to collectively regulate gut microbiota and ameliorate gut microbiota dysbiosis, yielding a synergistic therapeutic impact on colitis. This work offers an oral probiotic delivery strategy that enhances the functional properties and viability of probiotics within the gastrointestinal tract, while also introducing a natural therapeutic concept utilizing a bacteria-microalgae symbiotic system for the management of gastrointestinal disorders.

## Materials and methods

### Probiotics and microalgae culture experiments

Strain EcN, a kind gift of Professor Jinyao Liu (Shanghai, China), was cultivated in lysogeny broth (LB) culture media in a 250-mL Erlenmeyer flask at 37 °C and 200 rpm. 10 g tryptone, 5 g yeast extract, and 10 g NaCl were included in one liter of LB, and the initial pH of the media was 7.4. EcN samples were collected at 12 h cultivation, and harvested by centrifugation (6,500 ×g, 10 min, 4 °C), then washed using phosphate buffered saline (PBS) three times, and the resulting cells were resuspended with PBS (OD_600_ = 1). SP and its culture media, Zarrouk media, were both purchased from Guangyu Biological Technology (Shanghai, China). SP was cultured in an illumination incubator (Bluepard, Shanghai, China) at 30 °C. One liter of Zarrouk media contained 16.8 g NaHCO_3_, 2.5 g NaNO_3_, 1 g NaCl, 0.5 g K_2_HPO_4_, 1 g K_2_SO_4_, 0.2 g MgSO_4_, 0.01 g FeSO_4_·7H_2_O, 0.08 g EDTA, 0.04 g CaCl_2_, 0.00286 g H_3_BO_3_, 0.00186 g MnCl_2_, 0.00022 g ZnSO_4_, 0.00008 g CuSO_4_, and 0.00005 g Co(NO_3_)_2_·6H_2_O. SP samples were harvested by centrifugation (3,260 ×g, 10 min, 4 °C), and washed by PBS three times, and the cells were resuspended with PBS (OD_680_ = 1). EcN-SP was obtained by adding equal volume of bacterial samples into SP solution.

### Characterization of EcN, SP, and EcN-SP

Strain EcN was first stained using the Gram stain method, and then using an optical microscope (Zeiss, Oberkochen, Germany) to capture bright-field and fluorescence microscope images. The morphology of EcN, SP, and EcN-SP was measured using a scanning electron microscope (SEM, SU-8010, Hitachi, Japan). Firstly, the samples were fixed with 2.5% glutaraldehyde in phosphate buffer (0.1 M, pH 7.0) overnight. and washed three times using phosphate buffer (0.1 M, pH 7.0). To protect the surface structure of EcN-SP during the subsequent processing, the sample was placed on a square filter paper and folded into 1 cm^2^ after pouring out the glutaraldehyde. All samples were fixed with 1% OsO_4_ in phosphate buffer for 2 hours and washed by phosphate buffer for three times. Then, the samples were sequentially dehydrated in 30, 50, 70, 80, 90, 95, and 100% ethanol. Finally, the samples were completely dehydrated using a Hitachi HCP-2 critical point dryer. The samples were covered with gold-palladium in a Hitachi model E-1010 ion sputter and observed using an SEM.

### Coculture of EcN with SP in the light or dark conditions

To explore the effect of SP and the SP extract after ultrasonic decomposition (SP-Ex) on the growth rate of strain EcN, EcN was cocultured with SP or SP-Ex in PBS with no nutrients. Before the experiment, the bacteria were freshly played on LB ager. Then, the single colonies of bacteria were picked and amplified in LB broth overnight (37 °C, 200 rpm). Strain EcN (OD_600_ = 2) and SP (OD_680_ = 2) were washed and harvested in sterile PBS as described above. SP was broken for 10 min using an ultrasonic crusher (ULD43, Ningbo Sincere Ultrasonic Equipment Technology Co. Ltd., Ningbo, China) at a sonication power of 75 W to obtain SP-Ex. EcN was mixed with SP or SP-Ex to achieved a 1:1 bacterium: microalgae ratio, and cocultured at 37 °C and 200 rpm for 8 h. Strain EcN was cultured alone with the same volume of PBS as a control. Samples were collected every 2 h and bacterial colony counts were performed using the spread plate counting method: samples were diluted for spread plate using LB agar media, and incubated at 37 °C for 24 h, the colonies on the plate were counted and analyzed. Furthermore, to investigate the effect of light on the proliferation of EcN in the symbiotic system, EcN and EcN-SP were cultured in PBS, simulated gastric fluid (SGF) containing pepsin (pH 2.0), and simulated intestinal fluid (SIF) with or without light. Samples from PBS were collected at 0, 2, 4, 6, and 8 h, from SGF at 0, 0.5, and 1 h, and from SIF at 0, 1, 2, 3, and 4 h. Bacterial colony counts were then performed as previously described, with colonies on the plates being counted and analyzed.

### Fluorescence imaging and in vivo biodistribution

All animal experiments and procedures in this study were approved and performed by the Institutional Animal Care and Use Committee of Zhejiang University. All animals in this study were purchased from Shanghai SLAC Laboratory Animal Co. Ltd. Mice were housed in isolator cages with sterilized bedding under the following auto-controlled conditions: temperature (24 °C), lighting (12 h light, 12 h dark), and relative humidity (55%).

Female C57BL/6 mice (six-week-old) were randomly allocated into two groups including EcN (10^8^ CFU EcN, 200 µL), and EcN-SP (10^8^ CFU EcN and 80 µg/mL SP suspensions, 200 µL). Mice were fasted for 12 h and orally administered with EcN and EcN-SP. After 1, 2, 4, 6, 8, and 24 h, the mice (*n* = 3) were sacrificed, and brain, heart, liver, spleen, lung, kidney, bladder, and GI tract of mice, were dissected and photographed with IVIS Lumina LT Series III (PerkinElmer, USA) using the fluorescence channel of GFP (excitation wavelength: 488 nm, emission wavelength: 505–550 nm) for EcN and chlorophyll (excitation wavelength: 605 nm, emission wavelength: 615–665 nm) for SP. The ileum (1 h after the oral administration) and colon (4 h after the oral administration) tissues in two groups were excised and incubated with PBS with 5% formaldehyde for fixing. Then, the samples were sectioned. Afterward, the samples were embedded in an OCT compound, and frozen. Frozen sections of ileum and colon were harvested with a slice thickness of 25 μm, and then the nuclei of the cells were stained using 4-6-diamidino-2-phenylindole (DAPI) for fluorescence microscope images.

### Cell culture

Normal rat small intestinal epithelial cells IEC-6 (ATCC; USA), were cultured in Dulbecco’s modified Eagle’s medium (DMEM) supplemented with 10% fetal bovine serum, 1% antibiotics, and 0.1 U/ml bovine insulin at 37 °C under 5% CO2 atmosphere.

### Cell counting kit-8 assay

IEC-6 cells were seeded into 96-well plates and cultured overnight. Afterward, IEC-6 cells were incubated with SP and SP-Ex at a series concentration (0, 1.6, 3.2, 6.3, 12.5, 25, and 50 µg/mL) for 24 h, respectively. The IEC-6 cell viability after dealing with SP or SP-Ex were verified by using cell counting kit-8 (CCK-8) assay (Beyotime, China). The absorbance at 450 nm was measured by using a microplate reader to verify the cell viability.

### Assessment of the anti-inflammatory effect of SP and SP-Ex

IEC-6 cells were seeded and incubated into 24-well plates overnight. Then the cells were incubated with 50 µg/mL lipopolysaccharide (LPS) and 50 µg/mL SP or SP-Ex for 12 h to test the anti-inflammatory effect of SP and SP-Ex. DCFH-DA assay kit (YEASEN, Shanghai, China) was used to measure the ROS generation. The images of treated cells were taken using a fluorescence microscope (Zeiss, Oberkochen, Germany), and then the fluorescent signals were quantified by using a flow cytometer (Beckman, California, USA) for further verification.

### Anti-inflammatory effects against DSS-induced mouse colitis

After acclimation, six-week-old female C57BL/6 mice were randomly divided into five groups (*n* = 6): 1, PBS (normal control, 200 µL PBS); 2, DSS + PBS (200 µL PBS); 3, DSS + EcN (10^8^ CFU EcN, 200 µL/mouse); 4, DSS + SP (80 µg/mL SP suspensions, 200 µL/mouse); and 5, DSS + EcN-SP (10^8^ CFU EcN and 80 µg/mL SP suspensions, 200 µL/mouse). The acute colitis of mice in the 4 treatment groups (PBS, DSS + PBS, DSS + EcN, DSS + SP, DSS + EcN-SP) were induced by providing drinking water containing DSS (3%, w/v) for the first 7 days. Mice were orally administered with PBS, EcN, SP, or EcN-SP every other day during the 2 weeks, respectively. Daily clinical assessment of DSS-induced colitis, including measuring body weight and noting rectal bleeding, stool conditions, and blood in stool using the disease activity index (DAI) scoring system was performed. Mice from different groups were sacrificed on the 14th day to dissect colons of each mouse. Then the length colons from different groups were measured, and the H&E, TNFα, and IL-6 staining were applied to the colons as well.

### 16 S rRNA sequencing analysis of gut microbiota

The feces of the mouse were collected on day 14 and stored at -80°C for further 16S rRNA gene sequencing. Magabio soil/fecal genomic DNA purification kit (Bioer, Hangzhou, China) was used to extract total genomic DNA. The concentration and purity were measured using the NanoDrop One (Thermo Fisher Scientific, MA, USA). Polymerase chain reaction amplification of the 16S rRNA gene V3-V4 region was conducted using the primer pairs F338 5’-ACTCCTACGGGAGGCAGCAG-3’ and R806 5’-GGACTACHVGGGTWTCTAAT-3’. Sequencing was performed on an Illumina Nova6000 platform by Guangdong Magigene Biotechnology Co., Ltd, China, and 250 bp paired-end reads were generated. OTUs were clustered in UPARSE [46]. Then, the taxonomy of the OTU representative sequence was assigned using the RDP classifier [47]. Further, the PICRUSt analysis workflow was applied to predict the functional composition of microbial communities’ metagenome from the 16 S profile [48]. Magichand Cloud Platform (http://www.magichand.online) was used to analyze all of the data.

### Biosafety assessment

To evaluate the biosafety of the bacteria-microalgae symbiotic system, twenty healthy female C57BL/6 mice were allocated into four groups (*n* = 6), and given PBS, EcN, SP, and EcN-SP respectively every three days for 30 days. During one month, the mice’s body weight was monitored, and then, the mice were sacrificed to harvest their blood samples and major organs for the hematological and pathological examinations after one month.

### Statistical analysis

All statistical analyses were conducted using Prism GraphPad v.8.00 (GraphPad Software, Inc., San Diego, CA, USA). In this study, all error bars were calculated from at least three independent experiments by using means ± SD. The *P* value indicates statistical significance as ns *p* > 0.05, *p < 0.05, **p < 0.01, ***p < 0.001.

## Results and discussion

### Construction and characterization of bacteria-microalgae symbiotic system (EcN-SP)

**Fig. 2:**
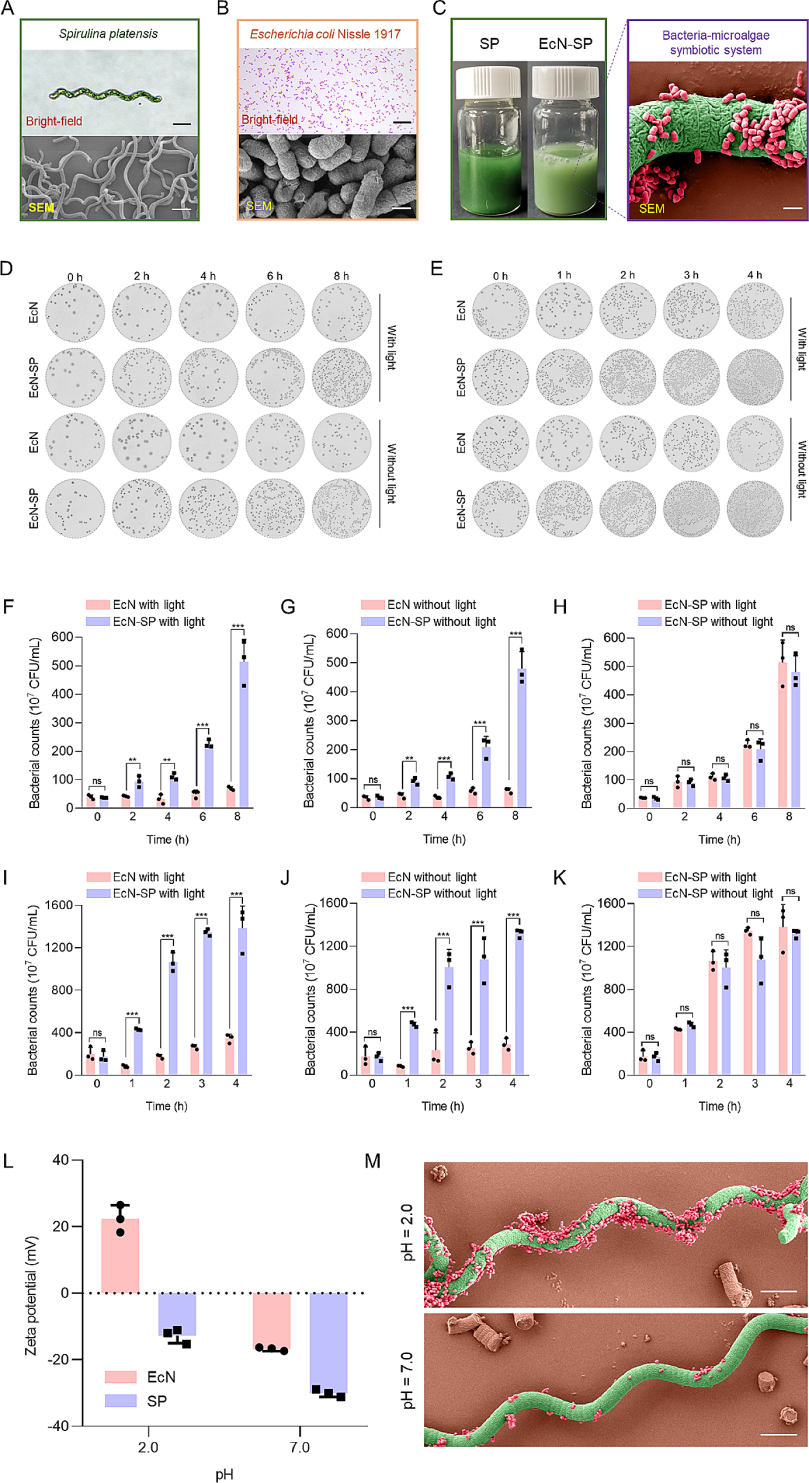
Construction and characterization of EcN-SP. (**A**) Bright-field and SEM images of SP (scale bar, 20 μm). (**B**) Bright-field image of Gram stained EcN (scale bar, 20 μm) and SEM image of EcN (scale bar, 500 nm). (**C**) Photograph of SP and EcN-SP samples (left side view). Pseudo-color SEM image of EcN-SP (right side view). Green indicates SP and red indicates EcN (scale bar, 2 μm). (**D**) Typical image of LB agar plates used to determine colony counts of EcN and EcN-SP collected at 0, 2, 4, 6, and 8 h with or without light in PBS. (**E**) Typical image of LB agar plates used to determine colony counts of EcN and EcN-SP collected at 0, 1, 2, 3, and 4 h with or without light in SIF. (**F-H**) Bacterial counts of EcN and EcN-SP at 0, 2, 4, 6, and 8 h with or without light in PBS. (**I-K**) Bacterial counts of EcN and EcN-SP at 0, 1, 2, 3, and 4 h with or without light in SIF. The data show means ± SD. The *P* value indicates statistical significance determined using Student’s *t*-test (*n* = 3, ns *P* > 0.05, **P < 0.01, and ***P < 0.001). (**L**) Zeta potential of EcN and SP under pH 2.0 and 7.0. The data show means ± SD (*n* = 3). (**M**) Pseudo-color SEM image of EcN-SP under pH 2.0 and 7.0. Green indicates SP, and red indicates EcN (scale bar, 100 μm)

In this study, natural SP is utilized as a carrier for EcN delivery. The morphology, structure, and size of SP were analyzed through optical and scanning electron microscope (SEM) images, revealing a standard spiral shape with a length of approximately 100 μm (Fig. 2A). The EcN used in the study is a Gram-negative bacterium labeled with the GFP protein and is about 1–3 μm long and 0.5 μm wide (Fig. 2B). By employing a straightforward one-step mixing method, EcN could be effectively incorporated onto SP to create a bacteria-microalgae symbiotic system (EcN-SP). SEM image revealed a significant amount of EcN closely adhering to the surface of SP (Fig. 2C), suggesting a strong affinity between EcN and SP and the potential for probiotic delivery through a bacteria-algal symbiosis system. In addition, large-scale cultivation of SP can be easily carried out in a laboratory setting using a photobioreactor (Fig. S1). EcN growth is also rapid, entering the logarithmic phase about 2 h after the start of culture (Fig. S2). The low acquisition and cultivation costs of SP and EcN and the efficient construction of EcN-SP are conducive to promoting their productization and commercial transformation.

### SP carrier-promoted EcN proliferation and intestinal colonization

The proliferation of probiotics is critical for its intestinal colonization. SP, known for its high protein, polysaccharide, vitamin, and trace element content, could significantly promote the growth of EcN (Fig. 2D-K). The bacterial plate coating test results demonstrated that SP significantly promotes the proliferation of EcN in both light and dark environments, under PBS (Fig. 2D, F, G) and SIF (Fig. 2E, I, J) conditions. Additionally, the results indicated no significant difference in the bacterial counts of EcN in the EcN-SP symbiotic system, regardless of the presence or absence of light in PBS (Fig. 2D and H) and SIF (Fig. 2E and K). This suggests that the proliferation activity of EcN, as enhanced by SP, is not influenced by light conditions. Additionally, SP sonically decomposed derivative (SP-Ex) has a similar boosting effect (Fig. S3-4). These findings suggest that SP and its constituents can facilitate the proliferation of EcN, aiding in the enhancement of intestinal colonization and sustained functionality.

The acidic environment and presence of digestive enzymes in the stomach pose a threat to the survival and efficacy of probiotics, necessitating protective measures during gastric transit to address challenges related to reduced activity and compromised functionality. Notably, the zeta potential results showed that EcN exhibited a positive charge at pH 2.0 (representative of the gastric environment) and a negative charge at pH 7.0 (representative of the intestinal environment) (Fig. 2L). Conversely, the zeta potential of SP remained negative over the entire pH range (pH 2.0–10.0) (Fig. S5). We hypothesized that the surface charge variation characteristics of EcN and SP in different regions of the gastrointestinal tract may contribute to their tight binding in the stomach and their dissociation in the intestine. To investigate this hypothesis, we employed SEM to examine the morphological changes of EcN-SP at pH 2.0 (representative of the gastric environment) and pH 7.0 (representative of the intestinal environment). The results revealed that there were more EcN cells (red) attached to the SP (green) surface at pH 2.0 than pH 7.0 (Fig. 2M). Additionally, we investigated the protective effect of SP on bacteria EcN in simulated gastric fluid (SGF) under both light and dark conditions. The results showed that SP significantly improves the survival rate of EcN in SGF (Fig. S6-S8). Moreover, the presence or absence of light had no significant effect on SP’s ability to enhance EcN survival in gastric acid (Fig. S9). This suggests that SP likely protects EcN and facilitates its passage through the stomach by tightly binding to it, thereby improving the survival rate of EcN. The findings suggest that SP has potential as a carrier for the oral administration of probiotics, leveraging both its probiotic and surface properties to enhance probiotic proliferation, mitigate loss in the stomach, and facilitate colonization in the intestine.

### Fluorescence imaging-guided gastrointestinal distribution

**Fig. 3:**
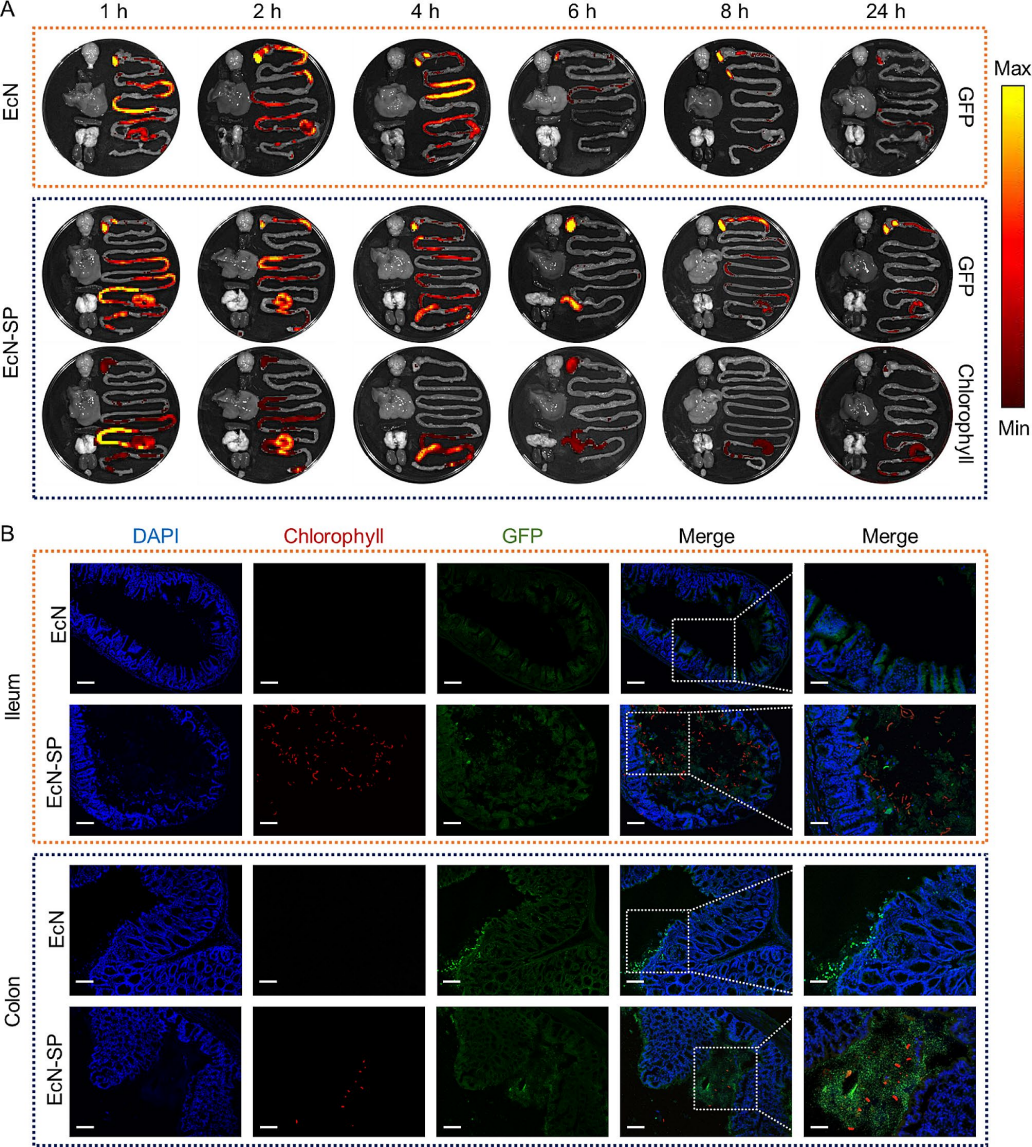
In vivo biodistribution. (**A**) Ex vivo fluorescence images of the GI tract and major organs, including brain, heart, liver, spleen, lung, kidney, and bladder collected at 1, 2, 4, 6, 8, and 24 h after the oral administration of EcN and EcN-SP, respectively. GFP channel: Ex, 488 nm; Em, 505–550 nm, and Chlorophyll channel: Ex, 605 nm; Em, 615–665 nm. (**B**) Fluorescence microscope images (blue, DAPI; green, GFP; red, chlorophyll) of frozen slices of ileum at 1 h and colon at 4 h after the oral administration of EcN and EcN-SP, respectively (scale bar, left,100 μm, right 50 μm)

The high chlorophyll content in SP results in the emission of red fluorescence when exposed to laser irradiation, while green fluorescent protein (GFP)-labeled EcN emits green fluorescence (Fig. S10). The distinct fluorescence properties of SP and EcN enable the visualization of their respective distributions using different fluorescence imaging channels. In order to investigate the potential enhancement of EcN colonization in the gastrointestine by SP carrier, in vivo fluorescence imaging was utilized to examine the distribution of EcN and EcN-SP in the gastrointestinal tract of mice following oral administration. As shown in Fig. 3A, EcN delivered by SP exhibited prolonged retention in the gastrointestinal tract compared to free EcN. Following oral administration of free EcN for 6 h, a notable decrease in GFP fluorescence signal was observed in the small intestine, with minimal signal remaining in the cecum and colon. Conversely, mice treated with EcN-SP displayed persistent GFP fluorescence signals in the cecum and colon 24 h post administration. Furthermore, co-localization of the chlorophyll signal with the EcN signal in EcN-SP-treated mice suggested dissociation of EcN from SP carriers and colonization of various regions within the intestine. The findings from frozen sections of the ileum and colon provided additional evidence to corroborate the aforementioned conclusions (Fig. 3B). Compared to direct oral administration of EcN, stronger green signals were observed in the ileum and colon of mice in the EcN-SP group, indicating a higher number of EcN cells in this group. These signals were distributed around the red signals, suggesting that the EcN cells were surrounded by SP. These results collectively indicate that the SP carrier enhances the retention and colonization of probiotics in intestinal tissues, and its fluorescent properties enable non-invasive monitoring of gastrointestinal distribution after oral administration.

### Anti-inflammatory effect of SP carrier

**Fig. 4:**
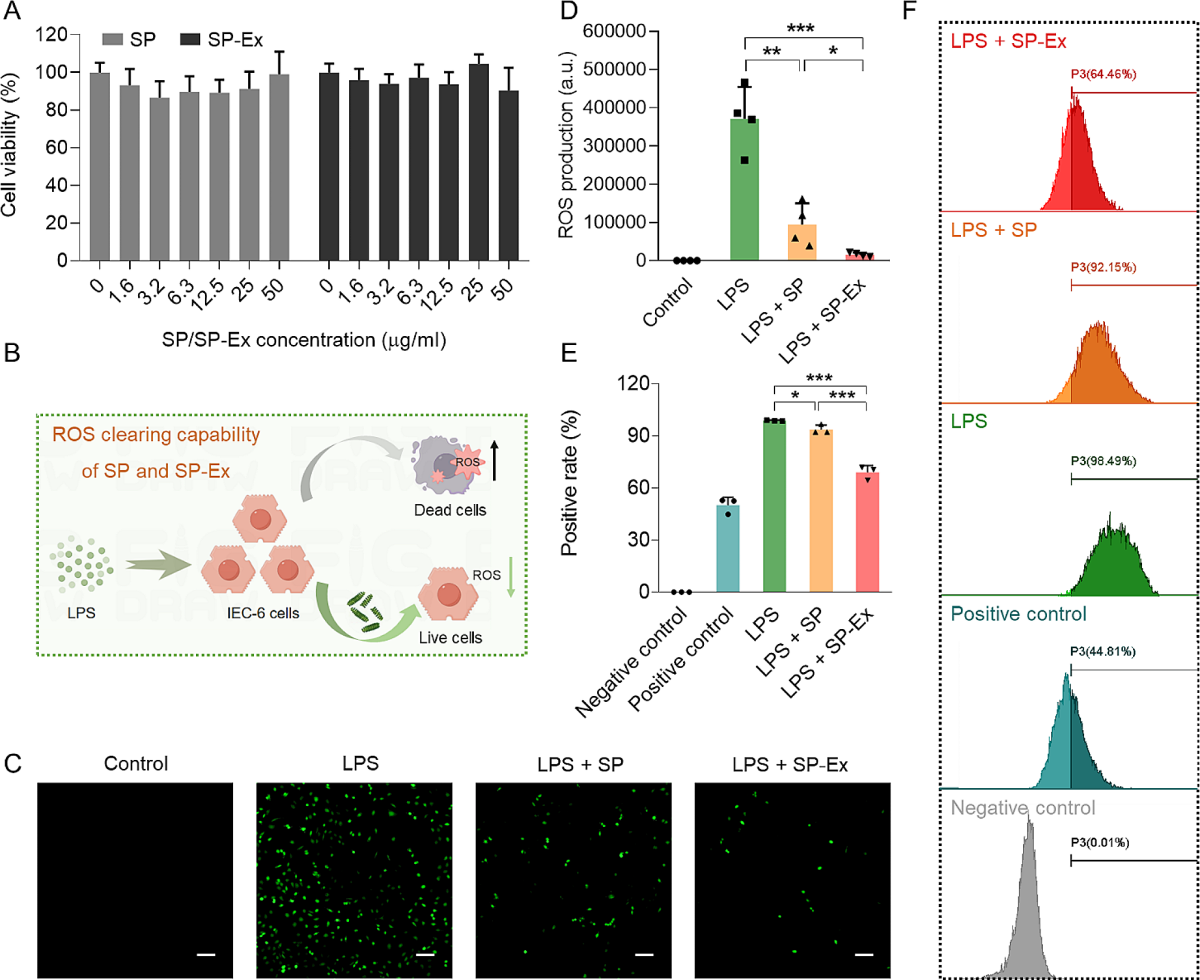
Anti-inflammatory effect of SP and SP-Ex. (**A**) Cell viabilities of IEC-6 cells after the incubation with different concentrations of SP or SP-Ex for 24 h, respectively. (**B**) Schematic illustration of ROS clearing capability of SP and SP-Ex in LPS-induced IEC-6 cell model. (**C**) DCFH-DA staining of ROS generation in IEC-6 cells after the incubation with 50 µg/mL LPS and 50 µg/mL SP or SP-Ex for 12 h. Scale bar, 100 μm. (**D**) Quantitative analysis of the fluorescence intensity of the generated ROS after different treatments. The data show means ± SD. The *P* value indicates statistical significance determined using Student’s *t*-test (*n* = 4, *P < 0.05, **P < 0.01, and ***P < 0.001). (**E**) Quantification of the ROS generation in IEC-6 cells after different treatments using flow cytometry. The data show means ± SD. The *P* value indicates statistical significance determined using Student’s *t*-test (n = 3, *P < 0.05, and ***P < 0.001). (**F**) Flow cytometry analysis showing the ROS generation in IEC-6 cells after different treatments

The oral safety and various active functions of SP as a dietary supplement have been demonstrated, including anti-inflammatory and antioxidant properties. In this study, we further investigated the in vitro biosafety and anti-inflammatory effects of SP on rat crypt epithelial cells (IEC-6). The cytotoxicity of SP and its extract (SP-Ex) was assessed using the CCK-8 method. Our results indicated that after co-incubation with all concentrations of SP and SP-Ex, the cell viability of IEC-6 cells was greater than 80%, suggesting that SP and its main components exhibited no significant toxic effects on IEC-6 cells (Fig. 4A). In order to examine the anti-inflammatory properties of SP and SP-Ex, we employed lipopolysaccharide (LPS) to induce the generation of reactive oxygen species (ROS) in IEC-6 cells, establishing an in vitro model of intestinal epithelial inflammation (Fig. 4B). The results of dichlorodihydrofluorescein diacetate (DCFH-DA) staining showed that LPS stimulated the production of ROS in IEC-6 cells (Fig. 4C). The intensity of green fluorescence indicative of ROS induced by LPS was notably diminished in the groups treated with SP or SP-Ex compared to the group treated solely with LPS (Fig. 4D). Flow cytometry analysis results provided additional evidence of the anti-inflammatory properties of SP and SP-Ex. The data indicated that both SP and SP-Ex were effective in reducing the production of ROS, with a particularly notable decrease in ROS-positive cell rates observed in the SP-Ex group, exceeding 30% (Fig. 4E and F). Overall, these in vitro findings support the conclusion that SP and its primary components exhibit favorable cytocompatibility and anti-inflammatory capabilities.

### Relieving effect of EcN-SP on intestinal inflammation in IBD

**Fig. 5:**
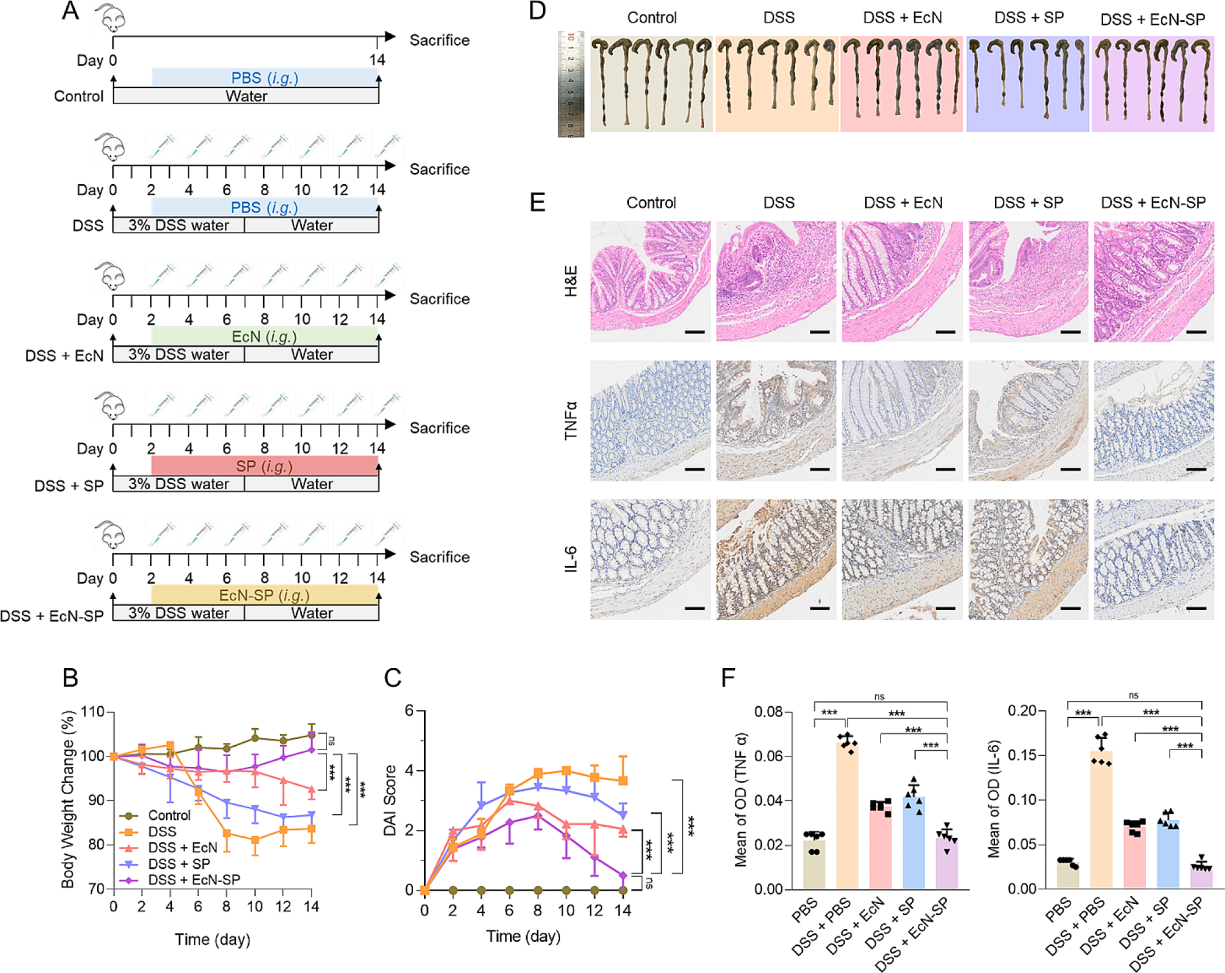
The therapeutic effect of EcN-SP in the DSS-induced colitis mice model. (**A**) Schematic illustration of the construction and treatment of DSS-induced colitis mice model. (**B**) Body weight change of the mice in different groups during the experiment. The data show means ± SD. The *P* value indicates statistical significance determined using Student’s *t*-test on day 14 (*n* = 6, ns *P* > 0.05, and ***P < 0.001). (**C**) Disease activity index (DAI) scores of the mice in different groups during the experiment. The data show means ± SD. The *P* value indicates statistical significance determined using Student’s *t*-test on day 14 (*n* = 6, ns *P* > 0.05, and ***P < 0.001). (**D**) Photographs of the colon appearance in different groups. (**E**) Represented H&E, TNFα, and IL-6 staining images of colons at day 15 in different groups (scale bar, 100 μm). (**F**) TNF-α and IL-6 positive intensity at day 15 in different groups. The data show means ± SD. The *P* value indicates statistical significance determined using Student’s *t*-test (*n* = 6, ns *P* > 0.05, and ***P < 0.001)

We subsequently established IBD model induced by dextran sulfate sodium (DSS) and assessed the in vivo anti-inflammatory effects of EcN-SP. Six-week-old female C57BL/6 mice were exposed to a 3% DSS drinking for 7 consecutive days and were intragastically administrated with PBS, EcN, SP, or EcN-SP every other day for a total of 7 doses (Fig. 5A). Throughout the treatment period, mice in the DSS group exhibited significant weight loss compared to the control group and showed a higher disease activity index (DAI), indicating the progression of DSS-induced ulcerative colitis (Fig. 5B and C). The EcN-SP group demonstrated a significant reversal of the weight loss and DAI increase induced by DSS. Additionally, both EcN and SP alone also showed a certain therapeutic effect, albeit significantly lower than the EcN-SP group. Following a two-week treatment period, the mice were euthanized and colon tissues were collected for macroscopic and microscopic examination. Compared to the control group, the DSS group displayed significant symptoms of colon shortening, whereas treatment with EcN-SP significantly restored colon length to normal levels (Fig. 5D and Fig. S11). Hematoxylin and eosin (H&E) staining revealed that EcN-SP treatment mitigated histological damage induced by DSS, preserving the structural integrity of the colon epithelium and crypts (Fig. 5E). Immunohistochemical analysis demonstrated a marked increase in the expression of pro-inflammatory factors, including tumor necrosis factor α (TNF-α) and interleukin-6 (IL-6), in the DSS group, whereas EcN-SP treatment significantly attenuated the expression of these pro-inflammatory factors (Fig. 5E and F). The findings indicate that EcN-SP may mitigate DSS-induced ulcerative colitis in mice through preservation of intestinal epithelial integrity and function, as well as suppression of pro-inflammatory factors production.

### Regulation effect of EcN-SP on gut microbiota

**Fig. 6:**
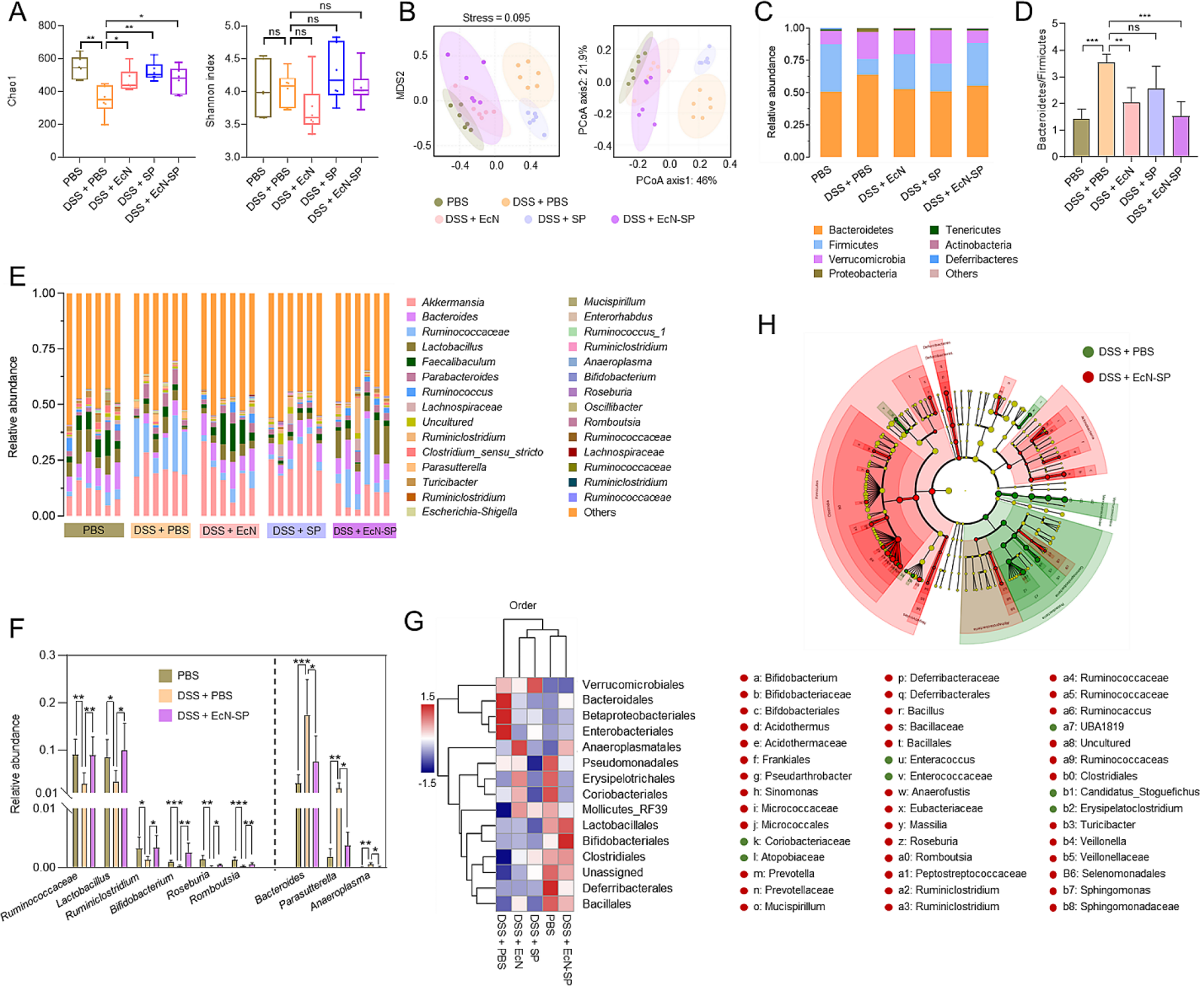
16 S rRNA sequencing analysis of gut microbiota regulated by EcN-SP. (**A**) Chao1 and Shannon index showing the community richness and the community diversity, respectively. The data show means ± SD. The *P* value indicates statistical significance determined using Student’s *t*-test (*n* = 6, ns *P* > 0.05, *P < 0.05, and **P < 0.01). (**B**) Non-metric multidimensional scaling (NMDS) and principal coordinate analysis (PCoA) showing bacterial community structures in different groups (*n* = 6, the dotted circle represents the 95% confidence interval). (**C**) Relative abundance of the bacterial community at the phylum level in different groups. (**D**) Bacteroidetes/Firmicutes ratio of the different groups. The data show means ± SD. The *P* value indicates statistical significance determined using Student’s *t*-test (*n* = 6, ns *P* > 0.05, **P < 0.01, and ***P < 0.001). (**E**) Relative abundance of the bacterial community at the genus level and each column represents each mouse (*n* = 6). (**F**) Represented probiotics and harmful bacteria in gut microbiota for PBS, DSS + PBS, and DSS + EcN-SP. The data show means ± SD. The *P* value indicates statistical significance determined using Student’s *t*-test (*n* = 6, *P < 0.05, **P < 0.01, and ***P < 0.001). (**G**) Hierarchically clustered heatmap of bacterial distribution of different communities from the different groups at the order level. Row represents the Z values obtained after normalization of relative abundances of each bacterial order, and column stands for different groups. The Z values for each bacterial order were depicted by color intensity with the legend indicated at the left of the figure. (**H**) LEfSe taxonomic cladogram of DSS + PBS, and DSS + EcN-SP showing the difference in taxa. The dot size is proportional to taxon abundance

To explore the impact of the EcN-SP on gut microbiota, fecal samples from all experimental groups were collected for 16 S rRNA sequencing analysis. The results of alpha (α)-diversity analysis revealed a significant reduction in Chao1 and community richness following DSS treatment, which was subsequently restored with various therapeutic interventions (Fig. 6A and Fig. S12A). However, our analysis revealed no statistically significant differences in the Shannon and Simpson diversity indexes when comparing community diversity among the five groups (Fig. 6A and Fig. S12B). Additionally, the beta (β)-diversity of microbial communities (NMDS and PCoA) based on the operational taxonomic units (OTUs) data from fecal samples subjected to various treatments were analyzed (Fig. 6B). The results showed that DSS treatment had a significant impact on the composition of gut microbiota, suggesting a restructuring of the bacterial community. Interestingly, the groups treated with DSS + EcN and DSS + EcN-SP exhibited greater similarity to the PBS group than the DSS + PBS and DSS + SP groups. These results indicate that EcN-SP has the potential to enhance the recovery of bacterial community diversity and composition within the gut microbiota.

Taxonomic classification of all operational taxonomic units (OTUs) was conducted at various levels, from phylum to genus. The results revealed that Bacteroidetes and Firmicutes were the dominant phyla (Fig. 6C). The Bacteroidetes/Firmicutes ratio, a key indicator of IBD, was observed to increase in the DSS + PBS group, but was significantly reduced to normal levels in the DSS + EcN-SP group (Fig. 6D). Moreover, at the phylum level, the presence of Proteobacteria decreased while Tenericutes and Actinobacteria increased in response to EcN-SP compared to the DSS + PBS group (Fig. 6C). Additionally, the relative abundance of probiotics such as *Ruminococcaceae_UCG-014*, *Lactobacillus*, *Ruminiclostridium_9*, *Bifidobacterium*, *Roseburia*, and *Romboutsia* at the genus level significantly decreased in the DSS + PBS group but returned to normal levels in the DSS + EcN-SP group. On the contrary, the presence of harmful bacteria such as *Bacteroides*, *Parasutterella*, and *Anaeroplasma* was found to be significantly elevated following DSS treatment, whereas a decrease in these bacteria was observed in the DSS + EcN-SP group (Fig. 6E and F). Similarly, the analysis of microbial community composition at various taxonomic levels (class, order, and family levels) revealed a consistent pattern of abundance in both beneficial and harmful bacteria in the DSS and EcN-SP treatment groups (Fig. S13-S16). Furthermore, hierarchically clustered heatmaps of bacterial distribution indicated that the DSS + EcN-SP group exhibited a closer resemblance to the PBS group compared to other treatment groups (Fig. 6G and Fig. S17-S19), indicating the protective effect of the EcN-SP on the gut microbiota. Subsequently, a linear discriminant analysis (LDA) effect size (LEfSe) was conducted to detect potential biomarkers at varying levels. The LEfSe analysis revealed 43 and 16 bacterial taxa exhibiting significant distinctions in the DSS + PBS and DSS + EcN-SP groups, respectively (Fig. 6H). The relative abundance of these biomarkers signifies notable variances and responses to EcN-SP (Fig. S20). These findings imply that DSS administration can substantially modify the composition of gut microbiota, while treatment with EcN-SP may aid in its restoration.

Subsequently, we employed Clusters of Orthologous Groups (COG) and Kyoto Encyclopedia of Genes and Genomes (KEGG) functional annotation and prediction to investigate the functional changes in gut microbiota across various groups. According to COG annotation, EcN-SP exhibited advantageous functions related to transcription, replication, recombination, repair, and signal transduction mechanisms compared to the DSS group (Fig. S21 and Fig. S22). Additionally, through KEGG annotation and enrichment analysis, the DSS + PBS group was effectively differentiated from the DSS + EcN-SP group (Fig. S23). The disparities between these groups primarily centered on metabolic pathways as revealed by the KEGG pathway enrichment analysis (Fig. S24). These results suggest that the effects of DSS on gut microbiota functionality can be altered and partially restored through EcN-SP treatment.

### Long-term oral biosafety of EcN-SP

**Fig. 7:**
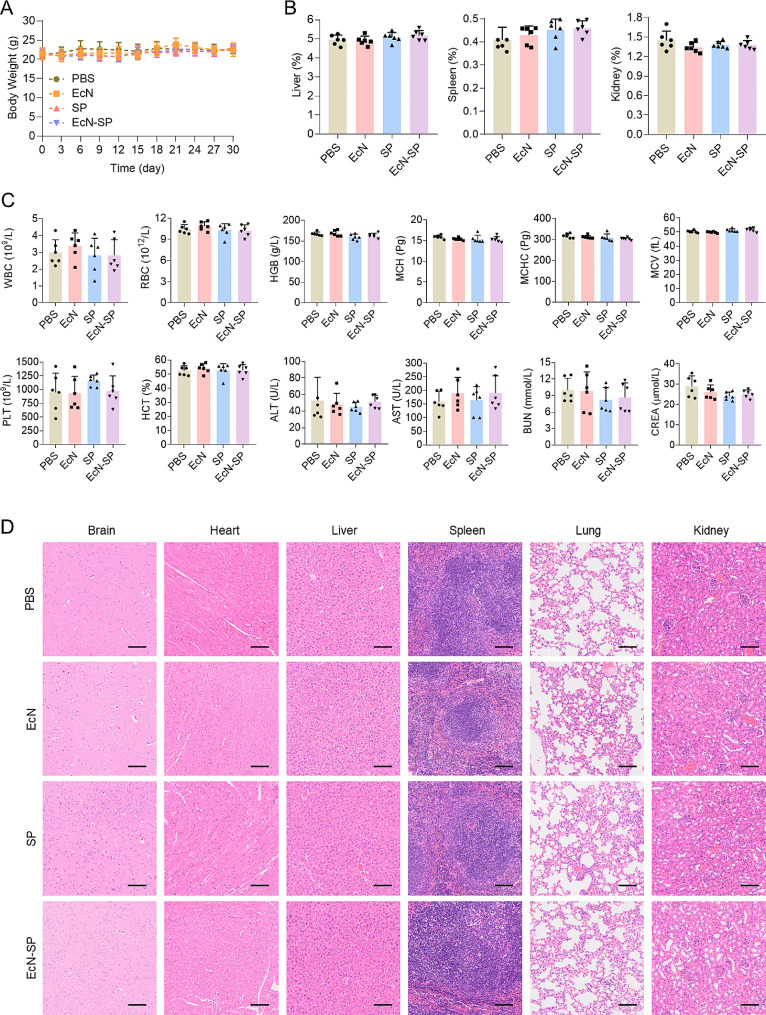
Long-term oral biosafety of EcN-SP. (**A**) Body weight of the mice in different groups during the experiment. The data show means ± SD (*n* = 6). (**B**) Organ coefficients (liver, spleen, and kidney) of the mice after every three days’ administration of PBS, EcN, SP, and EcN-SP for 30 days. The data show means ± SD (*n* = 6). (**C**) The long-term biosafety of EcN-SP. Haematological and biochemical analysis of the mice after every three days’ administration of PBS, EcN, SP, and EcN-SP for 30 days. The data show means ± SD (*n* = 6). WBC, white blood cells; RBC, red blood cells; HGB, hemoglobin; MCH, corpuscular hemoglobin; MCHC, corpuscular hemoglobin concentration; MCV, cell volume; PLT, blood platelet; HCT, hematocrit; ALT, alanine transferase; AST, aspartate transferase; BUN, blood urea nitrogen; CREA, creatinine. (**D**) Represented H&E staining images of the major organs (brain, heart, liver, spleen, lung, and kidney) of the mice after every three days’ administration of PBS, EcN, SP, and EcN-SP for 30 days (*n* = 6)

Finally, the long-term oral biosafety of EcN-SP was investigated. Over a period of one month, mice were subjected to continuous administration of PBS, EcN, SP, and EcN-SP. The results indicated that there was no significant fluctuation in body weight among the four groups, and the average body weight did not differ significantly between the treatment and control groups (Fig. 7A). Following the treatments, blood and major organs of the mice were collected for further analysis. EcN, SP, and EcN-SP did not exhibit any deleterious effects on the organ coefficients (kidney, liver, and spleen) of mice (Fig. 7B). Additionally, the blood routine and blood biochemical indexes of mice in various treatment groups did not demonstrate significant variances compared to the control group (Fig. 7C). Furthermore, no inflammatory lesions were detected in the major organs (brain, heart, liver, spleen, lung, kidney) across different treatment groups (Fig. 7D). These findings suggest that EcN-SP does not induce significant blood and tissue toxicity following long-term oral exposure, thereby demonstrating favorable oral safety.

## Conclusion

SP is a species of cyanobacteria that has been extensively studied in pharmacological applications and the food industry [49]. Previous research has indicated that SP serves as a natural carrier for the effective loading and delivery of drugs, thereby enhancing the bioavailability and therapeutic effectiveness of pharmaceutical agents [30, 50]. In this study, we introduce a novel approach utilizing a bacteria-microalgae symbiosis system (EcN-SP) for the delivery of probiotics and facilitation of probiotic intestinal colonization (Fig. 1A). As a carrier for probiotics, SP offers several advantages: (1) functioning as a prebiotic to promote the proliferation of probiotics (Fig. 2D and E); (2) reducing the loss of probiotics through the stomach and prolong the retention time in the intestine (Figs. 2L and M and 3); (3) exhibiting anti-inflammatory properties and synergistically treat IBD with probiotics (Figs. 4 and 5); (4) regulating the gut microbiota and maintaining the gut microbiota homeostasis (Fig. 6); (5) demonstrating favorable oral safety with promising clinical applications (Fig. 7).

The construction of EcN-SP involves a straightforward combination of EcN and SP (Fig. 2A-C). Prior research has demonstrated that the extracellular products of SP have a notable impact on enhancing the proliferation of probiotics [51]. The addition of SP powder not only promotes the viability of bacteria but also prolongs the storage duration of yogurt [52]. Co-cultivation experiments have indicated that both SP and its powder have the ability to stimulate the growth of EcN (Fig. 2D and E). This suggests that SP may serve as a prebiotic in the transmission of bacteria-microalgae symbiotic systems, as previously documented [53]. Furthermore, zeta potential analyses indicate that pH levels can impact the surface charge characteristics of SP and EcN, consequently influencing the binding affinity of EcN-SP (Fig. 2L and M). Specifically, in the stomach environment with pH < 2.0, the binding strength between EcN and SP is enhanced, leading to a decreased loss of EcN within the gastric environment. Conversely, in the intestinal environment where pH above 2.0, the binding strength between EcN and SP is attenuated, facilitating the release and colonization of EcN in the intestine. The distinctive helical structure of the SP carrier, coupled with its robust motor capabilities, results in a more intricate contact with the intestine and facilitates attachment to the intestinal wall, thereby enabling prolonged retention within the intestine. Zhong et al. have illustrated that SP exhibits a higher propensity for adherence to intestinal villi compared to other spherical microalgae [30]. Consequently, the utilization of EcN-SP can extend the residence time of EcN in the intestine and enhance the efficacy of EcN’s therapeutic effects (Fig. 3A). This conclusion is supported by frozen tissue section images, which reveal a greater accumulation of EcN in the ileum and colon tissues of the EcN-SP group as opposed to the free EcN group (Fig. 3B).

SP demonstrates beneficial antioxidant and anti-inflammatory properties, effectively decreasing LPS-induced ROS accumulation in intestinal epithelial cells (Fig. 4). The administration of EcN-SP has been shown to alleviate colitis symptoms in IBD mouse model by mitigating intestinal inflammation and preserving the structural and functional integrity of the intestinal epithelium (Fig. 5). Furthermore, research suggests that SP may serve as a promising prebiotic, modulating gut microbiota and fostering the proliferation of beneficial probiotic organisms [54, 55]. Prior research has established that IBD can lead to disruption of the gut microbiota, a finding corroborated by the outcomes of 16 S rRNA gene sequencing analysis in the present study (Fig. 6). Previous studies have indicated that the elevated Bacteroidetes to Firmicutes ratio serves as a key marker of IBD, a trend also observed in our study. Nevertheless, our investigation revealed that treatment with EcN-SP notably decreased this ratio, indicating that the symbiotic relationship between bacteria and microalgae may ameliorate the severity of colitis. Furthermore, EcN-SP has been shown to enhance the presence of beneficial probiotics and decrease the prevalence of detrimental bacteria. This finding aligns with previous research [56–59], suggesting that EcN-SP possesses the ability to selectively modulate the gut microbiota. Additionally, EcN-SP was found to preserve a comparable microbial composition at various taxonomic levels when compared to the control group treated with PBS, indicating its potential to uphold gut microbial balance and mitigate inflammation. The analysis of gene function prediction through COG and KEGG pathways indicated that DSS-induced dysbiosis reduced the diversity of the gene function of gut microbiota, while treatment with EcN-SP was able to restore microbial genes and maintain bacterial balance. It is postulated that SP acts as a prebiotic that modulates the composition of gut microbiota, enhancing the regulatory effects of probiotic EcN.

Overall, the proposed bacteria-microalgae symbiosis system offers a promising and convenient method for delivering oral probiotics, suggesting a novel approach for probiotic therapy in the treatment of gastrointestinal diseases.

### Electronic supplementary material

Below is the link to the electronic supplementary material.

Supplementary Material 1

## Data Availability

No datasets were generated or analysed during the current study.
