# Supplementary material for: Breaking barriers: bacterial-microalgae symbiotic systems as a probiotic delivery system
Source: J Nanobiotechnology. 2024 Jun 25;22:371. doi: 10.1186/s12951-024-02647-6 (PMC11197275; doi:10.1186/s12951-024-02647-6)
Supplement: Supplementary file 1 — Supplementary Material 1 [file 12951_2024_2647_MOESM1_ESM.docx]

Supporting Information for

**Breaking barriers: bacterial-microalgae symbiotic systems as a probiotic delivery system**

*Hui Huang^1, 2^, Xiaoyang Liu^1, 2^, Yutong Lang^1, 3^, Jiarong Cui^1, 2^, Danni Zhong^1, 2^*, Min Zhou^1, 2, 3, 4^**

^1^Eye Center, the Second Affiliated Hospital, Zhejiang University School of Medicine, Hangzhou, 310009, China.

^2^Institute of Translational Medicine, Zhejiang University, Hangzhou, 310009, China

^3^Zhejiang University-University of Edinburgh Institute (ZJU-UoE Institute), Zhejiang University School of Medicine, Zhejiang University, Haining 314400, China

^4^State Key Laboratory (SKL) of Biobased Transportation Fuel Technology, Zhejiang University, Hangzhou, 310027, China

^∗^Correspondence: zhoum@zju.edu.cn (M. Z); 11718308@zju.edu.cn (D. Z).

**Supplementary Figures**


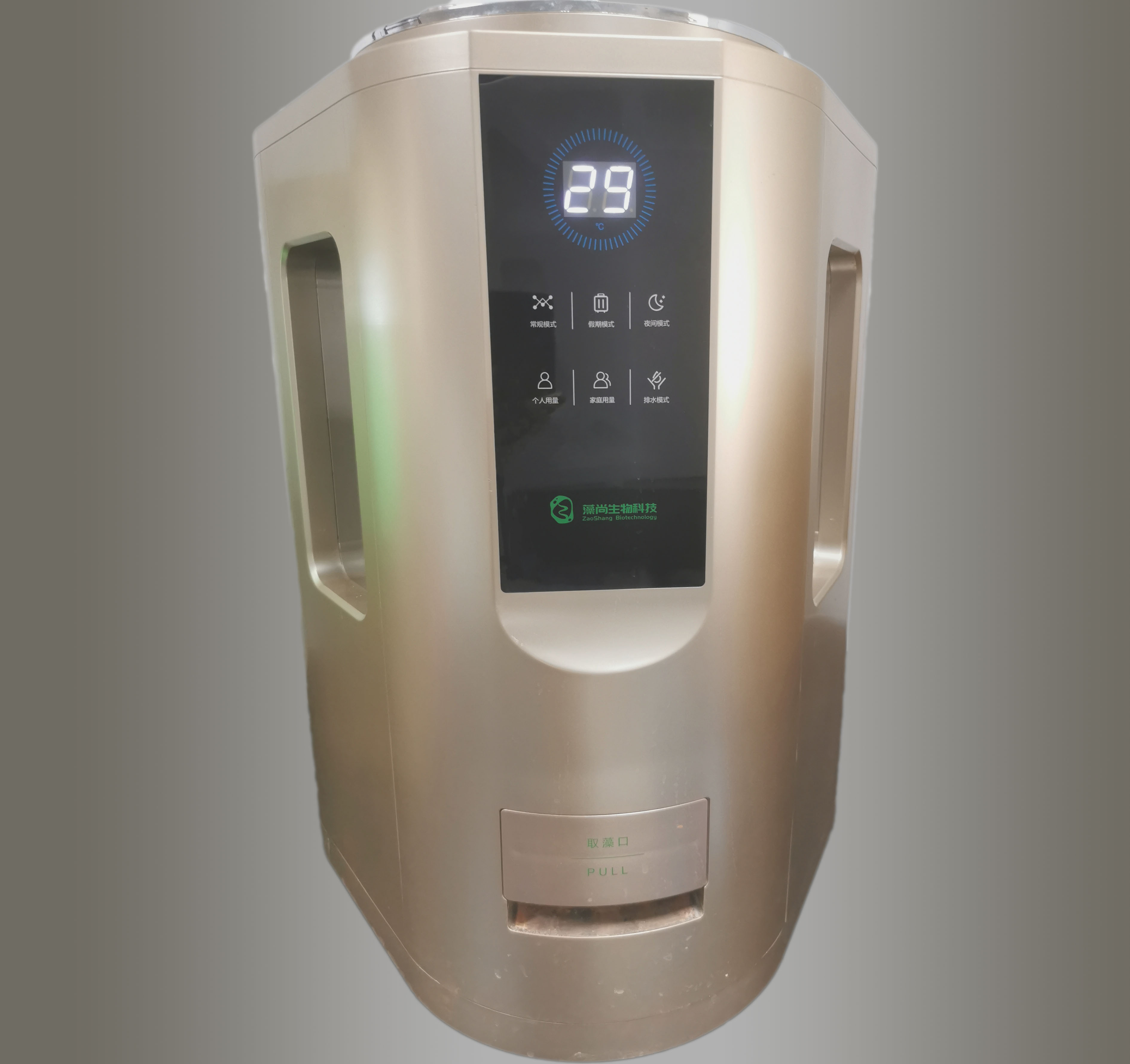


**Fig. S1** Photograph of the large-scale culture of SP in photobioreactor.


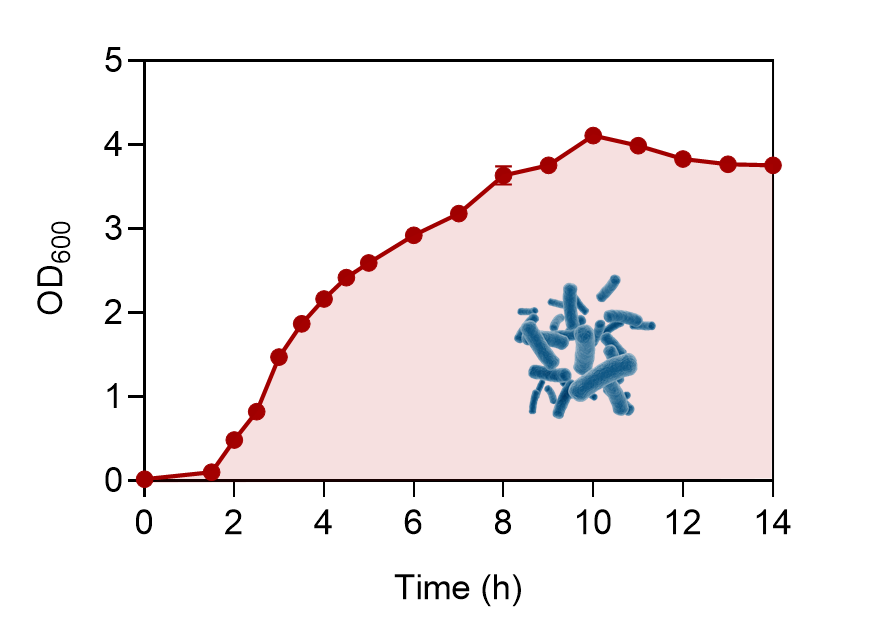


**Fig. S2** The growth curve of the strain EcN. The data show means ± SD (n = 3).


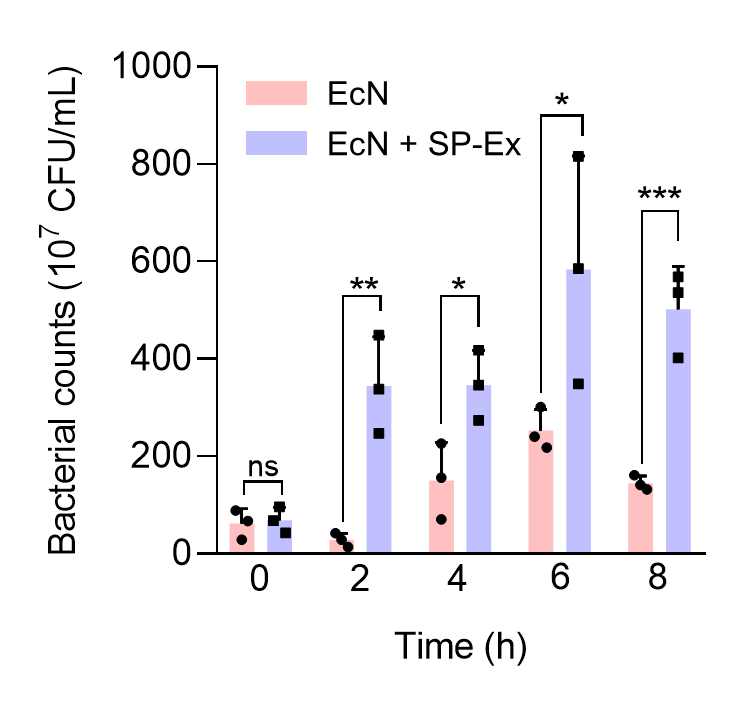


**Fig. S3** Bacterial counts of EcN cultured with or without SP-Ex at 0, 2, 4, 6, and 8 h. The data show means ± SD. The *P* value indicates statistical significance determined using Student’s *t*-test (n = 3, ns *P* > 0.05, **P* < 0.05, ***P* < 0.01, and ****P* < 0.001).


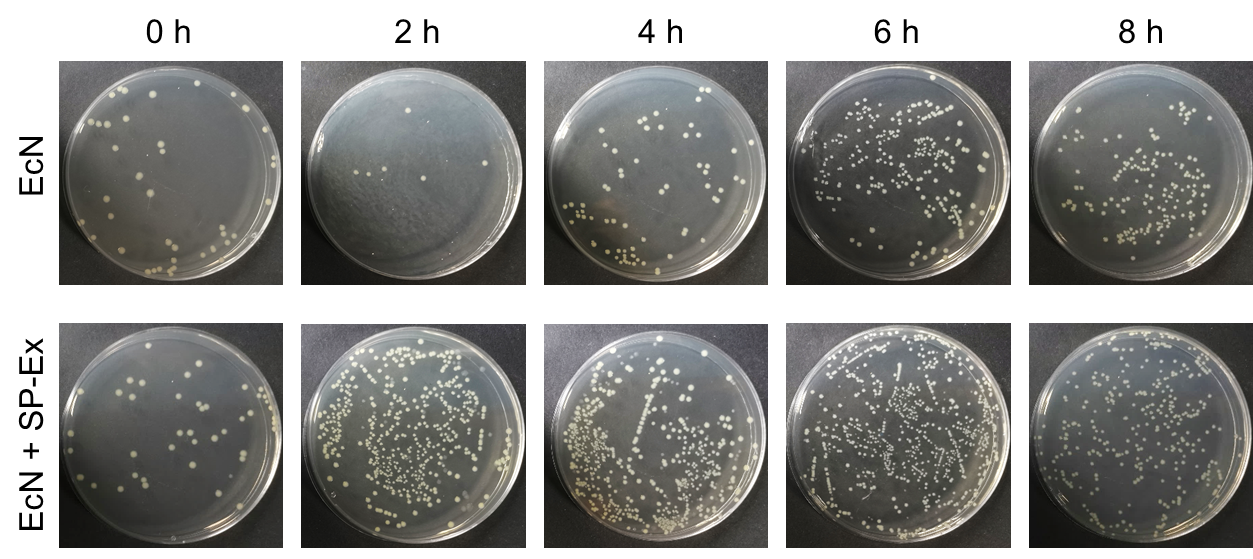


**Fig. S4** Bacterial typical spread plate of EcN cultured with or without SP-Ex at 0, 2, 4, 6, and 8 h.

**Fig. S5** Zeta potential of EcN and SP from pH 2.0 to 7.0. The data show means ± SD (n = 3).


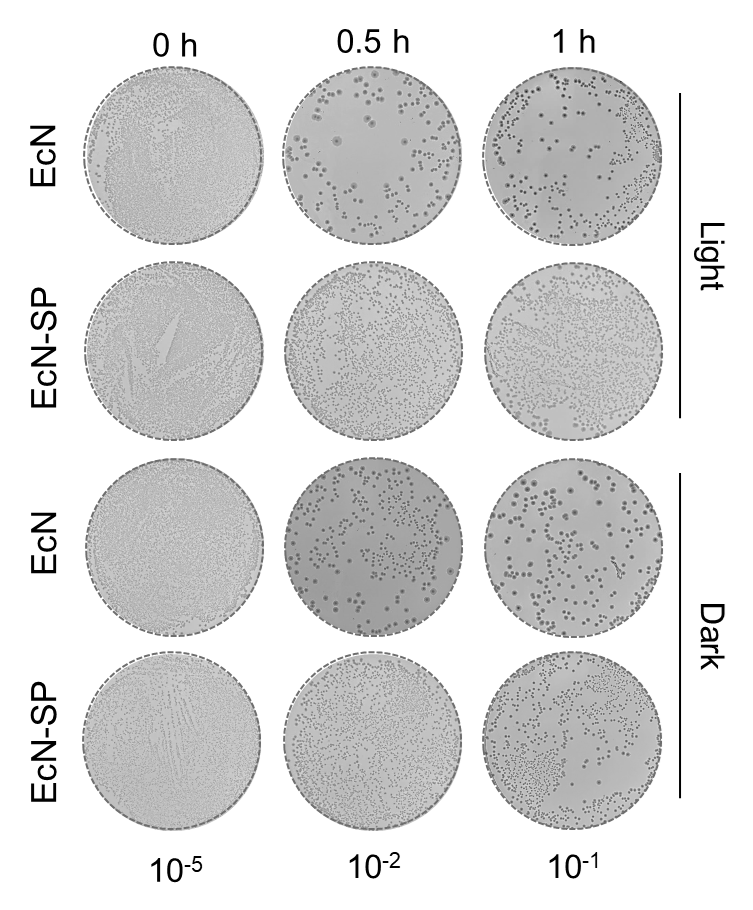


**Fig. S6** Typical image of LB agar plates used to determine the colony counts of EcN and EcN-SP collected at 0 (diluted 100,000 times), 0.5 (diluted 100 times), and 1 h (diluted 10 times) with or without light in SGF.


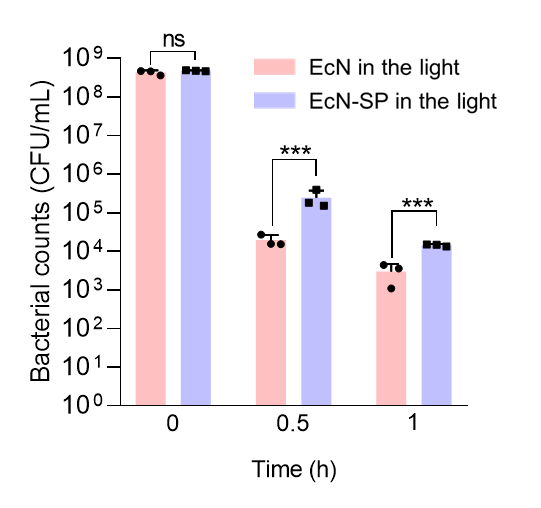


**Fig. S7** Bacterial counts of EcN and EcN-SP at 0, 0.5, and 1 h with light in SGF. The data show means ± SD. The *P* value indicates statistical significance determined using Student’s *t*-test (n = 3, ns *P* > 0.05, and ****P* < 0.001).


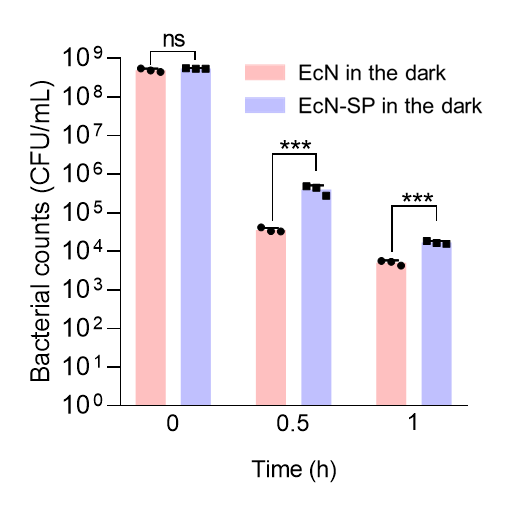


**Fig. S8** Bacterial counts of EcN and EcN-SP at 0, 0.5, and 1 h without light in SGF. The data show means ± SD. The *P* value indicates statistical significance determined using Student’s *t*-test (n = 3, ns *P* > 0.05, and ****P* < 0.001).


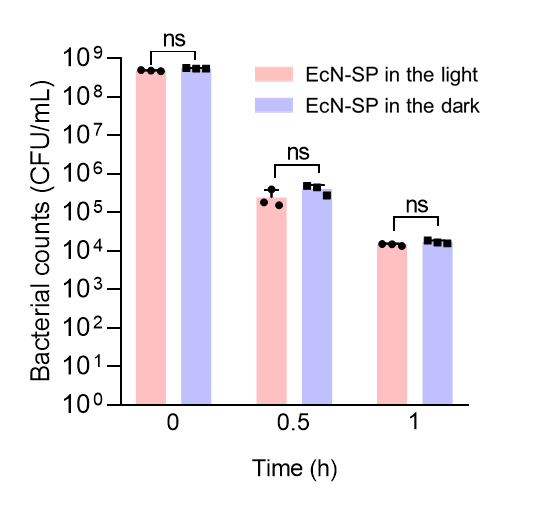


**Fig. S9** Bacterial counts of EcN-SP at 0, 0.5, and 1 h with or without light in SGF. The data show means ± SD. The *P* value indicates statistical significance determined using Student’s *t*-test (n = 3, ns *P* > 0.05).


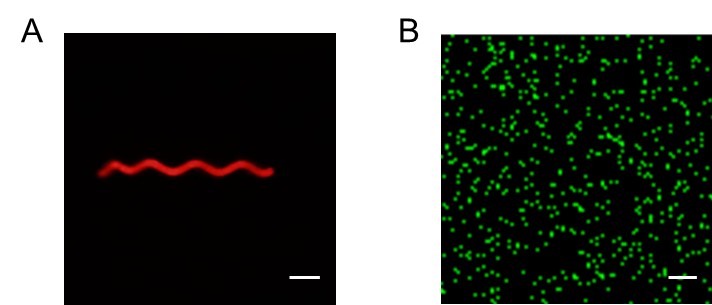


**Fig. S10** Fluorescence images of SP (scale bar, 20 μm) and GFP-labeled EcN (scale bar, 10 μm).

**Fig. S11** Colon length of the mice in different groups. The data show means ± SD. The *P* value indicates statistical significance determined using Student’s *t*-test (n = 6, ***P* < 0.01, and ****P* < 0.001).


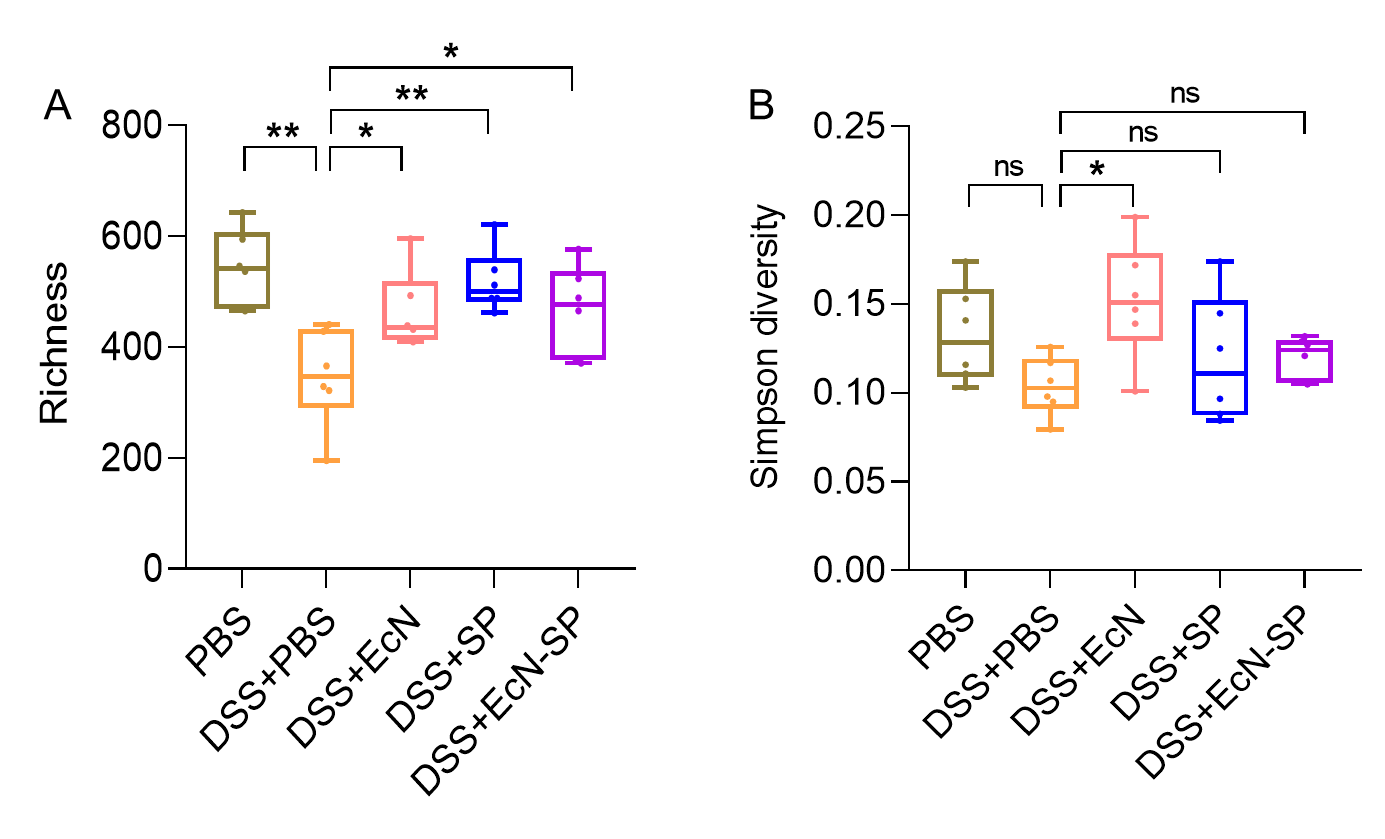


**Fig. S12** (A) Richness and (B) Simpson diversity of observed operational taxonomic units showed the α-diversity of the microbial community. The data show means ± SD. The *P* value indicates statistical significance determined using Student’s *t*-test (n = 6, ns *P* > 0.05, **P* < 0.05, and ***P* < 0.01).


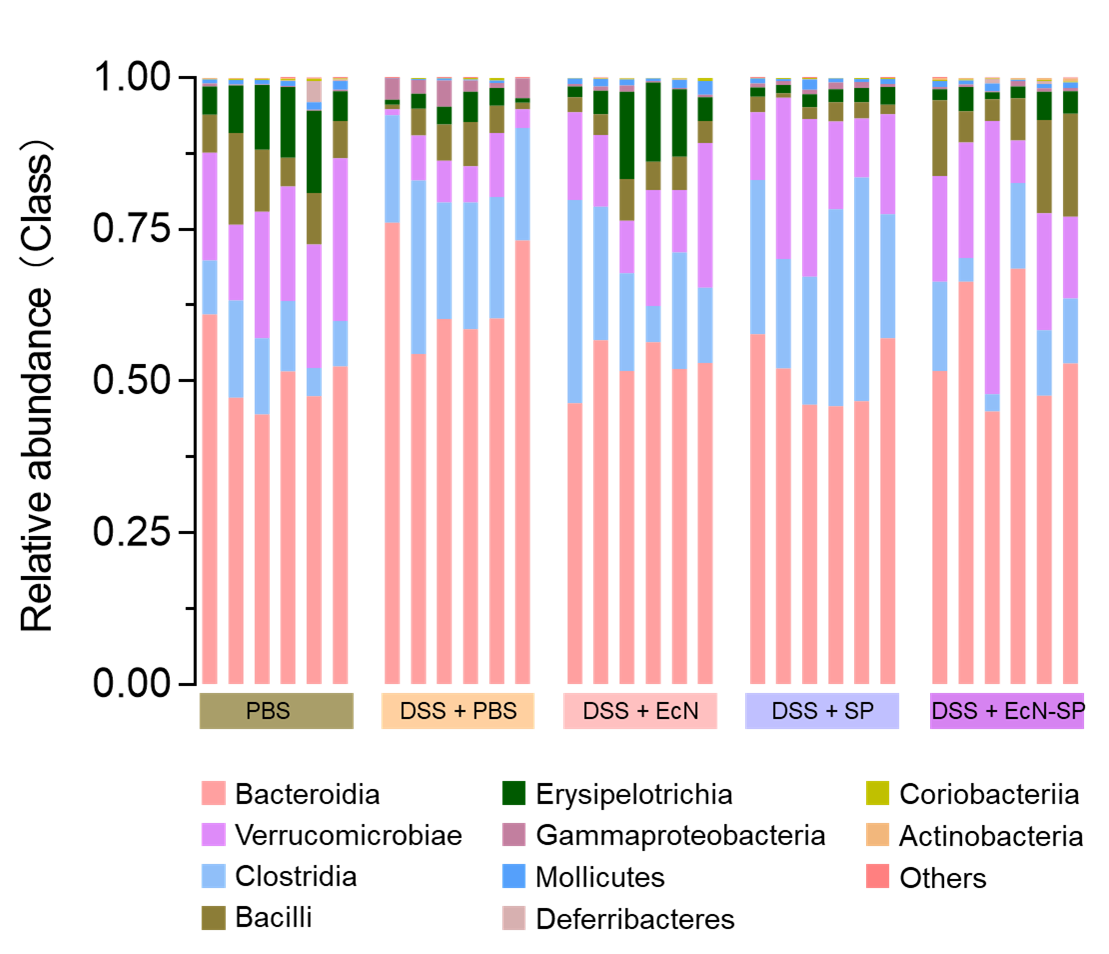


**Fig. S13** Relative abundance of the bacterial community at class level and each column represent each mouse (n = 6).


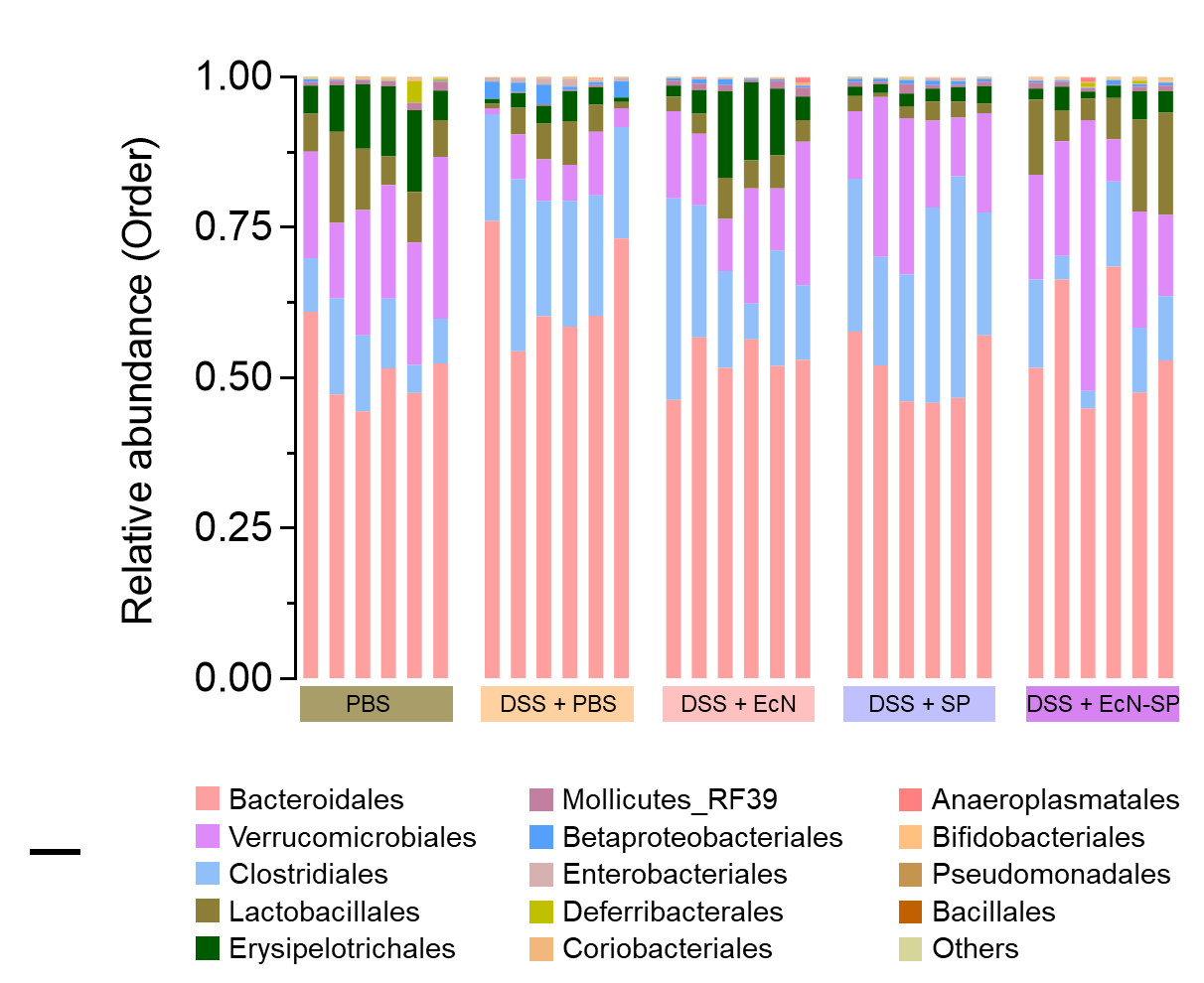


**Fig. S14** Relative abundance of the bacterial community at order level and each column represent each mouse (n = 6).


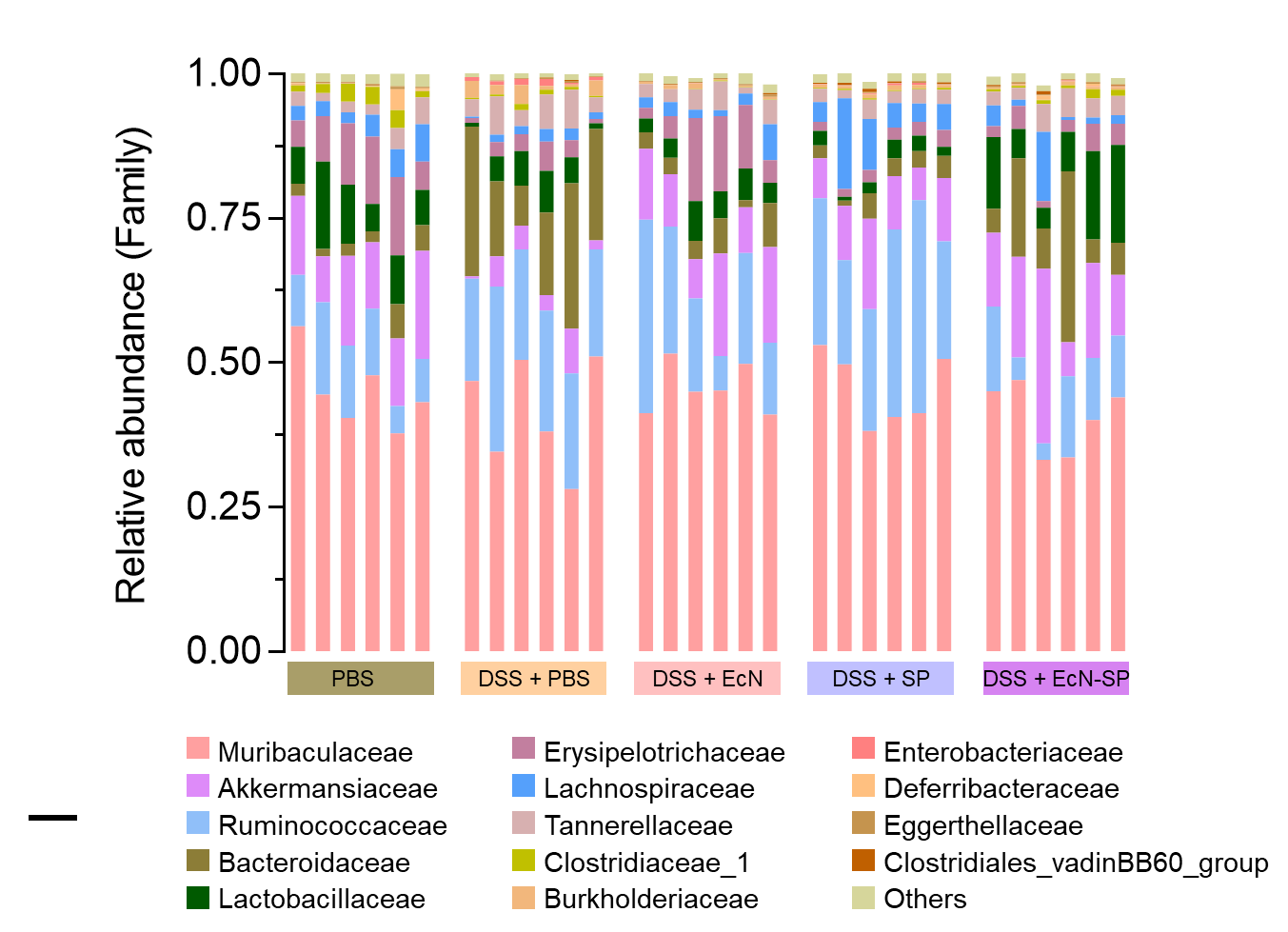


**Fig. S15** Relative abundance of the bacterial community at family level and each column represent each mouse (n = 6).


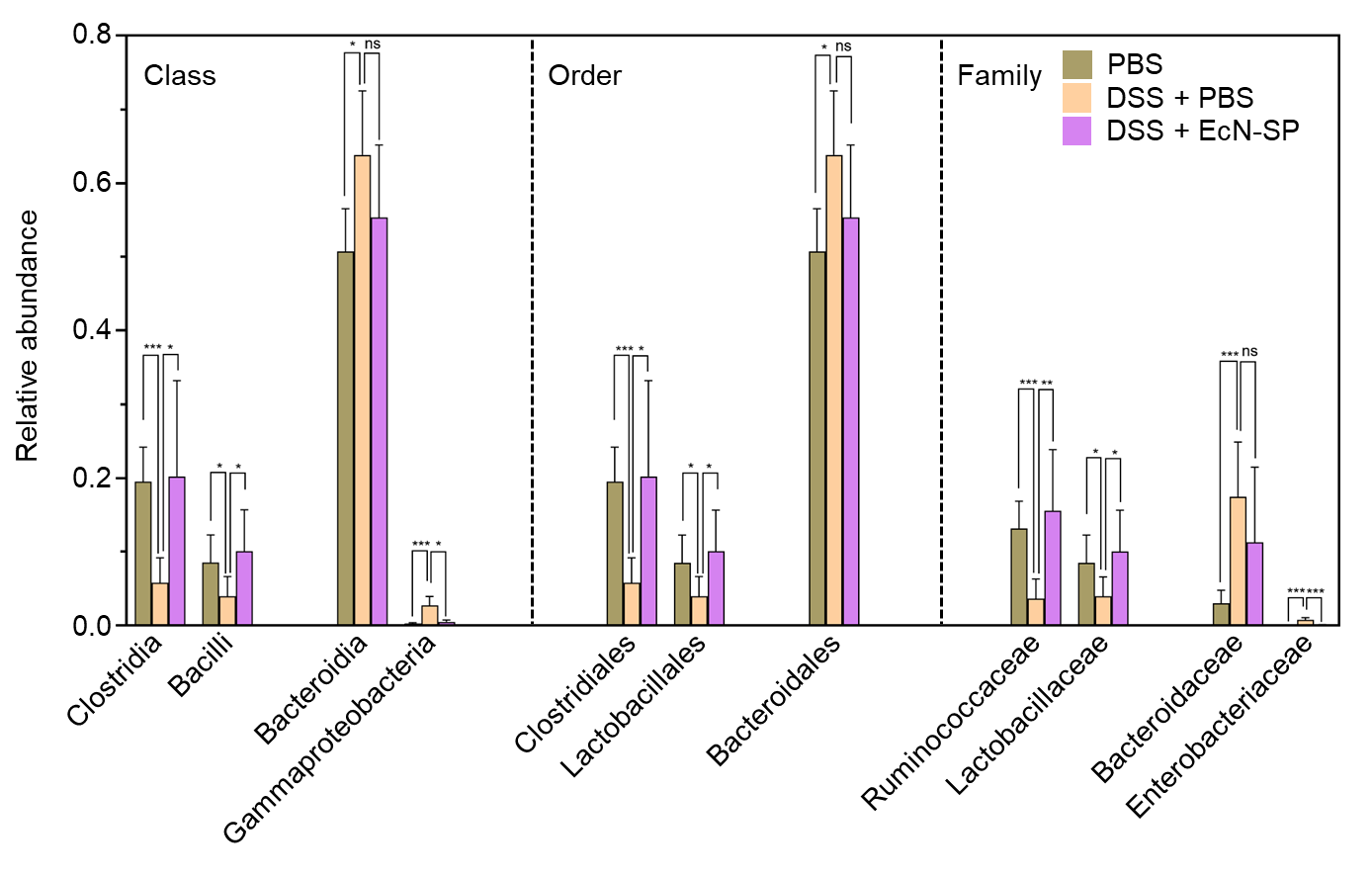


**Fig. S16** Represented probiotics and harmful bacteria in gut microbiota for PBS, DSS + PBS, and DSS + EcN-SP. The data show means ± SD. The *P* value indicates statistical significance determined using Student’s *t*-test (n = 6, ns *P* > 0.05, **P* < 0.05, ***P* < 0.01, and ****P* < 0.001).


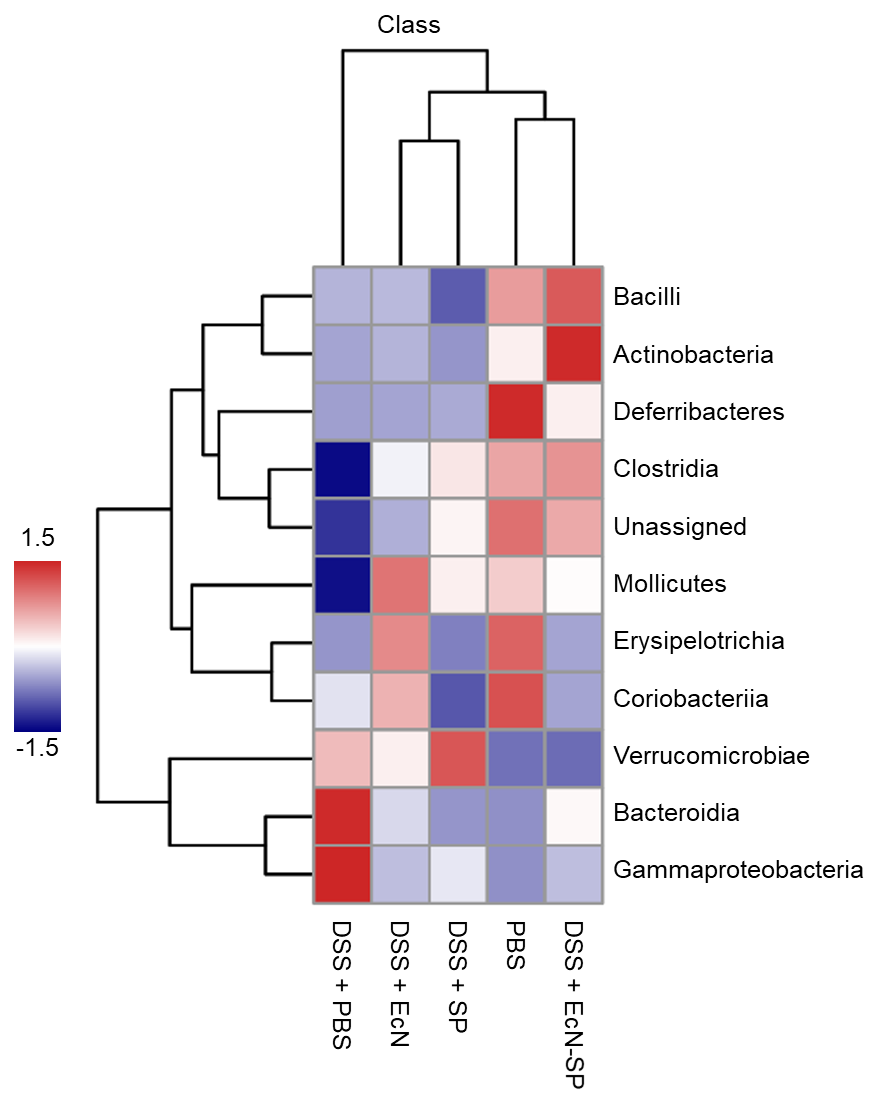


**Fig. S17** Hierarchically clustered heatmaps of bacterial distribution of different communities from the different groups at class level. Row represents the Z values obtained after normalization of relative abundances of each bacterial order, and column stands for different groups. The Z values for each bacterial class, family, and genus levels were depicted by color intensity with the legend indicated at the left of the figure.

**
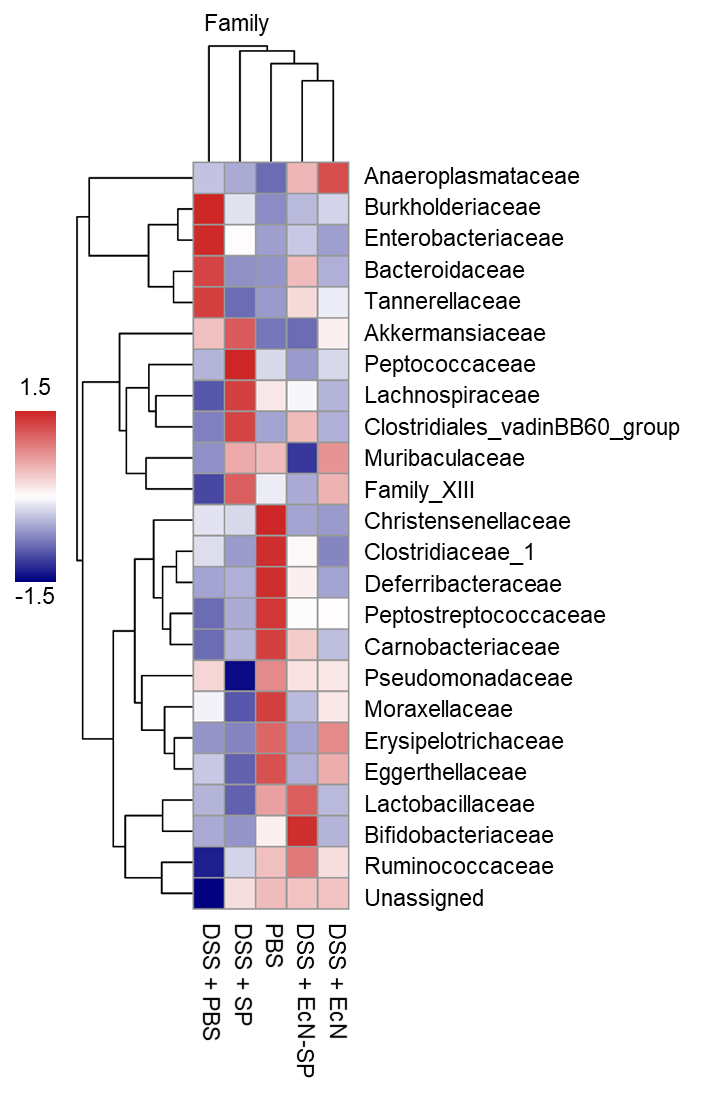
**

**Fig. S18** Hierarchically clustered heatmaps of bacterial distribution of different communities from the different groups at family level. Row represents the Z values obtained after normalization of relative abundances of each bacterial order, and column stands for different groups. The Z values for each bacterial class, family, and genus levels were depicted by color intensity with the legend indicated at the left of the figure.


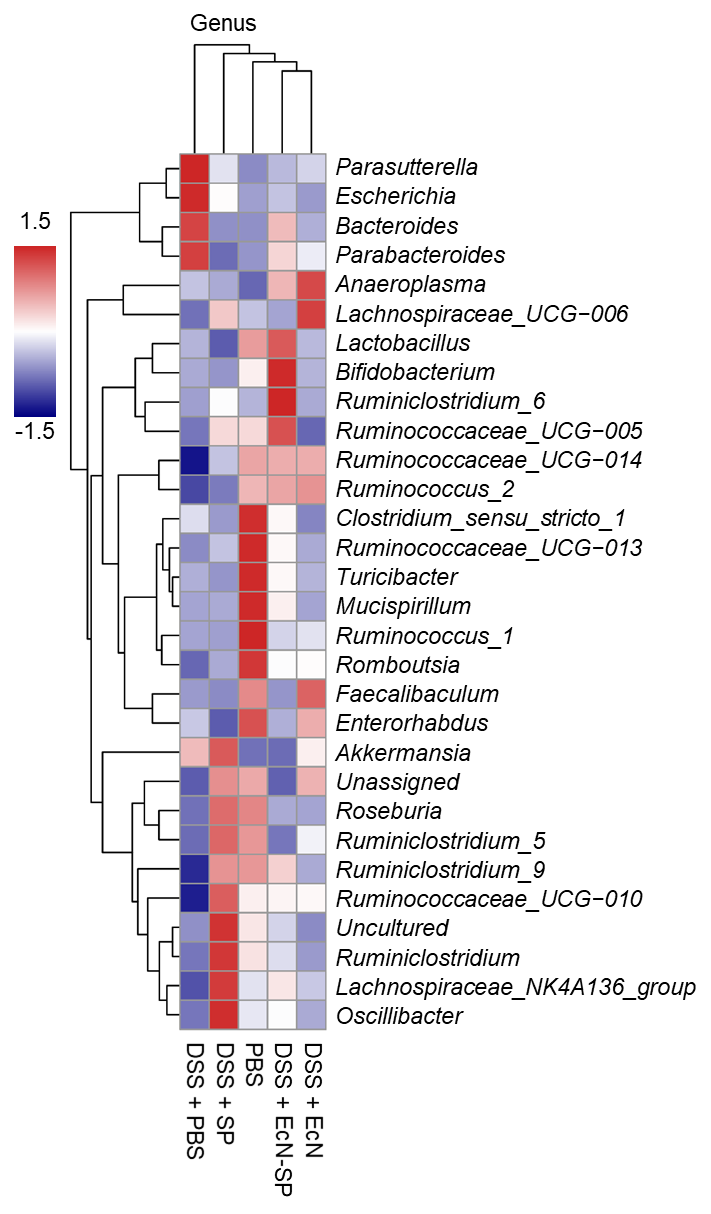


**Fig. S19** Hierarchically clustered heatmaps of bacterial distribution of different communities from the different groups at genus level. Row represents the Z values obtained after normalization of relative abundances of each bacterial order, and column stands for different groups. The Z values for each bacterial class, family, and genus levels were depicted by color intensity with the legend indicated at the left of the figure.


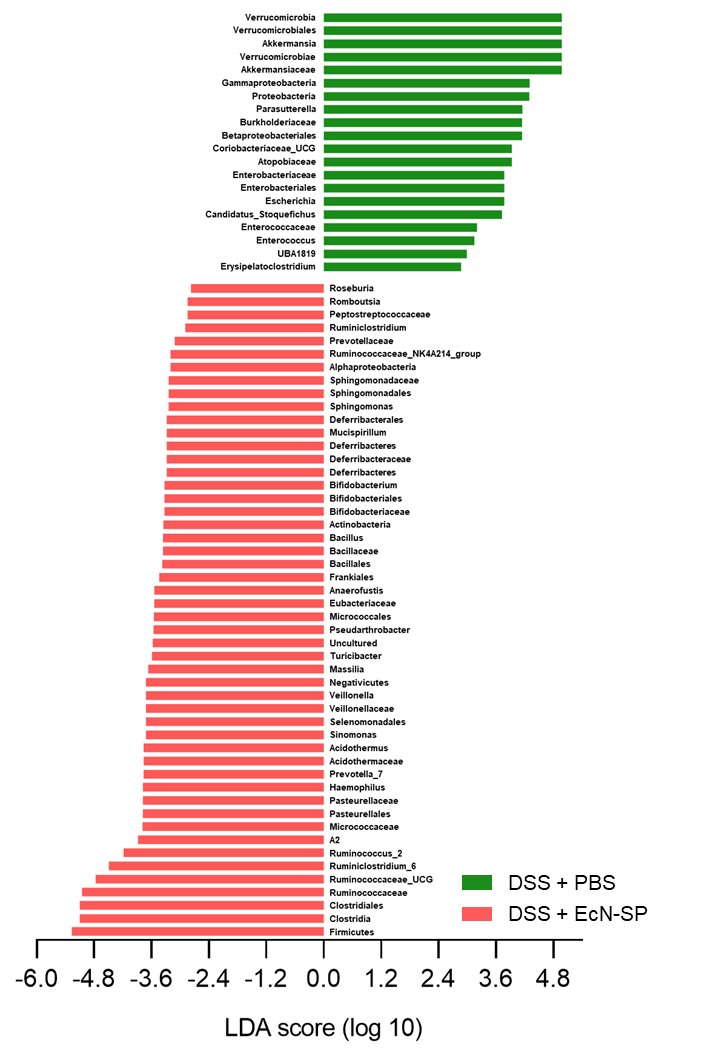


**Fig. S20** The discriminating bacteria among gut microbiota between DSS + PBS and DSS + EcN-SP groups.


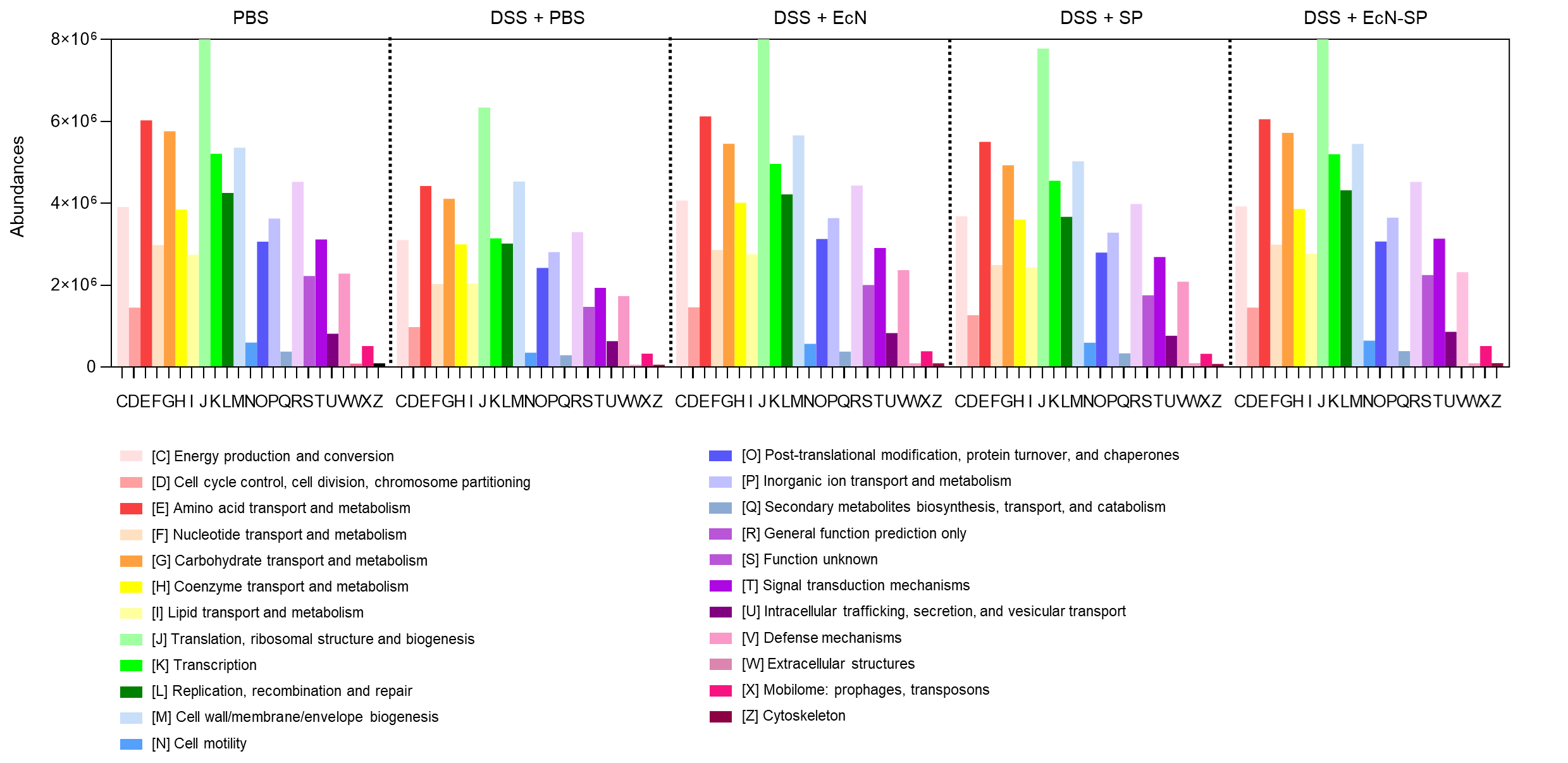


**Fig. S21** Abundances of clusters of orthologous groups (COG) categories in different groups.


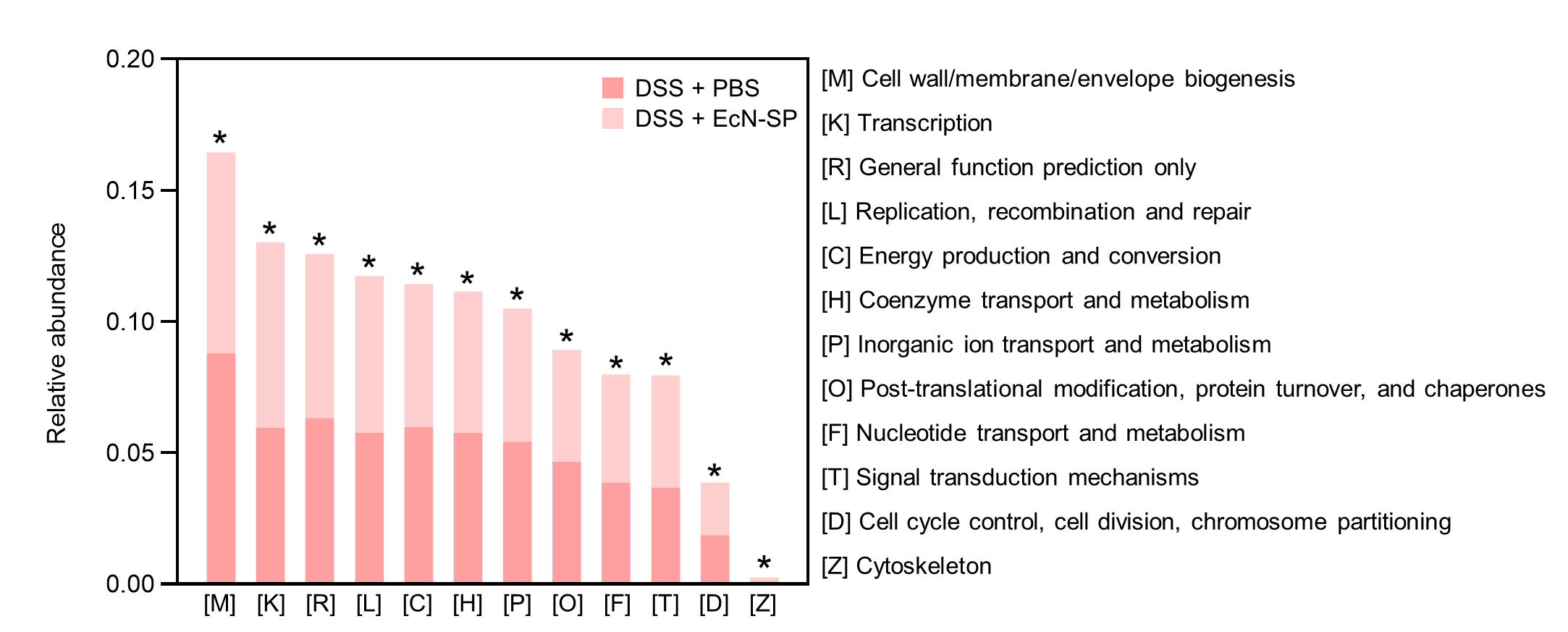


**Fig. S22** The different gene function analysis between DSS + PBS and DSS + EcN-SP groups based on COG.


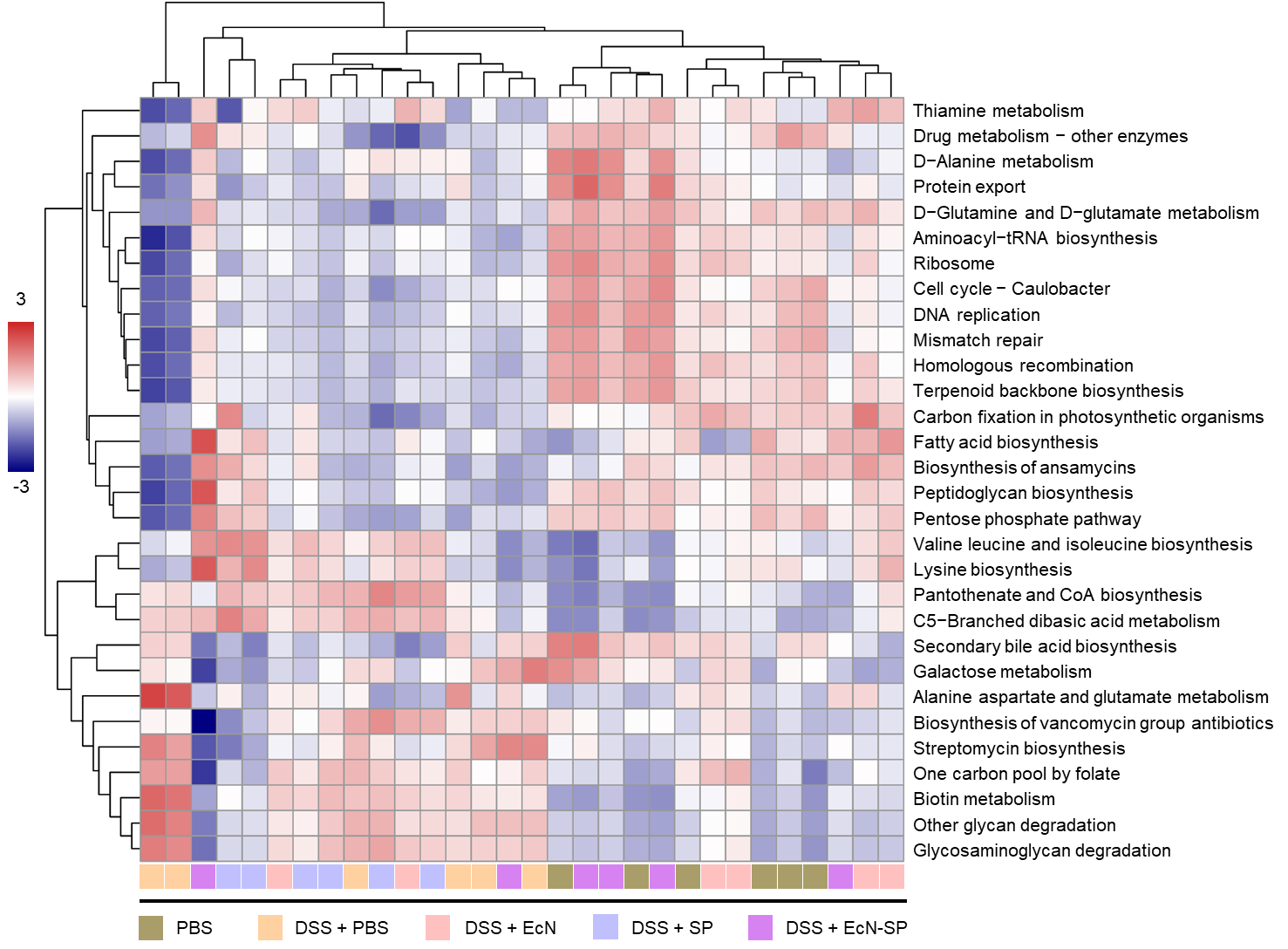


**Fig. S23** Kyoto encyclopedia of genes and genomes (KEGG) functional annotation and differential enrichment analysis in different groups.


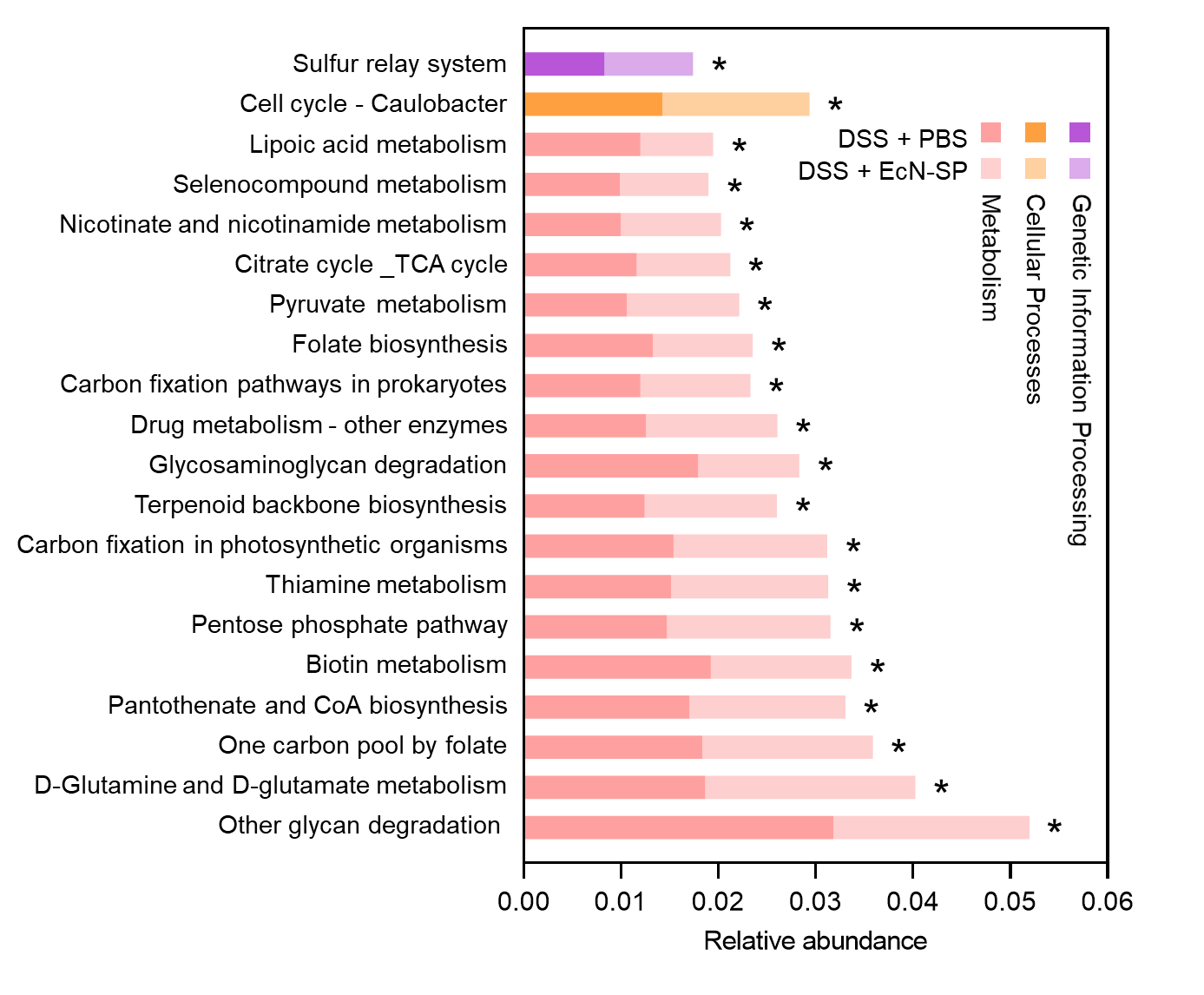


**Fig. S24** The different gene function analysis between DSS + PBS and DSS + EcN-SP groups based on KEGG pathway.
